# The association between Single Nucleotide Polymorphisms of Klotho Gene and Mortality in Elderly Men: The MrOS Sweden Study

**DOI:** 10.1038/s41598-020-66517-5

**Published:** 2020-06-24

**Authors:** Ping-Hsun Wu, Per-Anton Westerberg, Andreas Kindmark, Åsa Tivesten, Magnus K. Karlsson, Dan Mellström, Claes Ohlsson, Bengt Fellström, Torbjörn Linde, Östen Ljunggren

**Affiliations:** 1 Department of Medical Sciences, Uppsala University, Uppsala, Sweden; 2 Wallenberg Laboratory for Cardiovascular and Metabolic Research, Institute of Medicine, Sahlgrenska Academy, University of Gothenburg, Gothenburg, Sweden; 3 Clinical and Molecular Osteoporosis Research Unit, Department of Clinical Sciences and Orthopedic Surgery, Lund University, Skåne University Hospital, Lund, Sweden; 4 Center for Bone and Arthritis Research at the Sahlgrenska Academy, Institute of Medicine, Sahlgrenska Academy, University of Gothenburg, Gothenburg, Sweden; 5 Institute of Clinical Medicine, College of Medicine, Kaohsiung Medical University, Kaohsiung, Taiwan; 6 Faculty of Medicine, College of Medicine, Kaohsiung Medical University, Kaohsiung, Taiwan; 7 Division of Nephrology, Department of Internal Medicine, Kaohsiung Medical University Hospital, Kaohsiung, Taiwan

**Keywords:** Prognostic markers, Genetics research

## Abstract

The Klotho (*KL*) gene is involved in phosphate homeostasis. Polymorphisms in this gene have been reported to be associated with the risk of cardiovascular disease. Here we used computational tools to predict the damage-associated single nucleotide polymorphisms (SNPs) in the human *KL* gene. We further investigated the association of SNPs in the *KL* gene and mortality in the Swedish multicenter prospective Osteoporotic Fractures in Men (MrOS) cohort. This study included 2921 men (aged 69–81 years) with mean 4.49 ± 1.03 years follow-up. 18 SNPs in the *KL* gene were genotyped using Sequenom. These SNPs were identified by *in silico* tools for the coding and noncoding genome to predict the damaging SNPs. After quality analyses, SNPs were analyzed for mortality risk using two steps approach on logistic regression model screening and then Cox regression model confirmation. Two non-synonymous SNPs rs9536314 and rs9527025 were found to be potentially damaging SNPs that affect *KL* protein stability and expression. However, these two SNPs were not statistically significantly associated with all-cause mortality (crude Hazard ratio [HR] 1.72, 95% confidence interval [CI] 0.96–3.07 in rs9536314; crude HR 1.82, 95% CI 0.998–3.33 in rs9527025) or cardiovascular mortality (crude HR 1.52, 95% CI 0.56–4.14 in rs9536314; crude HR 1.54, 95% CI 0.55–4.33 in rs9527025) in additive model using Cox regression analysis. In conclusion, these two potentially damaging SNPs (rs9536314 and rs9527025) in the *KL* gene were not associated with all-cause mortality or cardiovascular mortality in MrOs cohort. Larger scales studies and meta-analysis are needed to confirm the correlation between polymorphisms of the *KL* gene and mortality.

## Introduction

The Klotho (*KL*) gene, which composed of five exons, encodes transmembrane protein type I (1014 and 1012 amino acids in human) that encompasses an extracellular domain with two internal repeats (KL1 and KL2), a membrane-spanning segment, and a short intracellular domain^1^. The *KL* protein, which is expressed predominantly in the distal tubules of the kidney, parathyroid cells, choroid plexus, and pituitary glands^1^, exists in both secreted and membrane-bound forms^2^. It is involved in longevity^1^, cardiovascular (CV) health^3^, and calcium and phosphorous regulation in the kidney^4^. Altered human *KL* expression is related to osteopenia/osteoporosis^5^, suppression of Wnt signaling^6^, amelioration of vascular endothelial dysfunction^7^, and in mediating the role of fibroblast growth factor 23 (FGF23) in bone-kidney-parathyroid control of phosphate and calcium^8^.

The human *KL* gene, located on chromosome 13q12, spans approximately 50 kb in length and contains 5 exons and 4 introns, encoding a 5.2 kb mRNA transcript^2^. Polymorphisms in the *KL* gene have been found associated with risk of coronary artery disease, stroke, and hypertension in different populations. A functional variant named *KL-VS*, which harbors two amino acid changes (F352V; rs9536314 and C370S; rs9527025), was shown to alter the number of extracellular levels of klotho and the ratio of intracellular vs. extracellular levels in transiently transfected HeLa cells^5^. This polymorphism was also associated with mortality risk among elderly individuals^5^, increased risk of coronary artery disease in the middle-aged population^9^, and related to lower high-density lipoprotein levels^3^, hypertension^3^, and stroke^3,10^ risk in the homozygous *KL-VS* genotype. However, some reports do not support the increased risk of coronary artery disease with the *KL-VS* genotype (rs9536314 and rs9527025)^11^. Further, only a few human exceptional longevity studies have examined *KL* variation and have published inconsistent results^5,12–14^. Therefore, the established relationships between *KL* SNPs and mortality or CV disease have lacked consistency. These studies are relatively small and require replication.

Beyond the coding genome, the non-coding genome also has regulatory elements driving gene expression^15^, such as gene promoters, enhancers, or binding sites for proteins or regulatory RNA. The intronic rs577912 SNP was found to be associated with mortality risk in patients receiving hemodialysis^16^. Furthermore, the CC genotype of this SNP was associated with a 16–21% lower Klotho expression compared with the AA/AC genotype when introduced into lymphoblastoid cell lines^16^. Since the *KL* gene is highly correlated to mortality and CV disease, complete evaluation of both coding and non-coding SNPs of the *KL* gene is important. Determining which SNPs affect the clinical phenotype would make it possible to identify the molecular mechanisms of disease variation. We hypothesized that variants in the *KL* gene might have an impact on mortality risk. The effects were evaluated by a combination of different approaches, including bioinformatics and clinical study. The aims of our study included a test in the *KL* gene polymorphisms using a computational approach based on tools to differentiate the deleterious or disease-associated SNPs first and then evaluate the clinical impact of selected *KL* gene polymorphisms on mortality in a cohort of elderly Swedish men.

## Methods

### Study design

**Figure 1:**
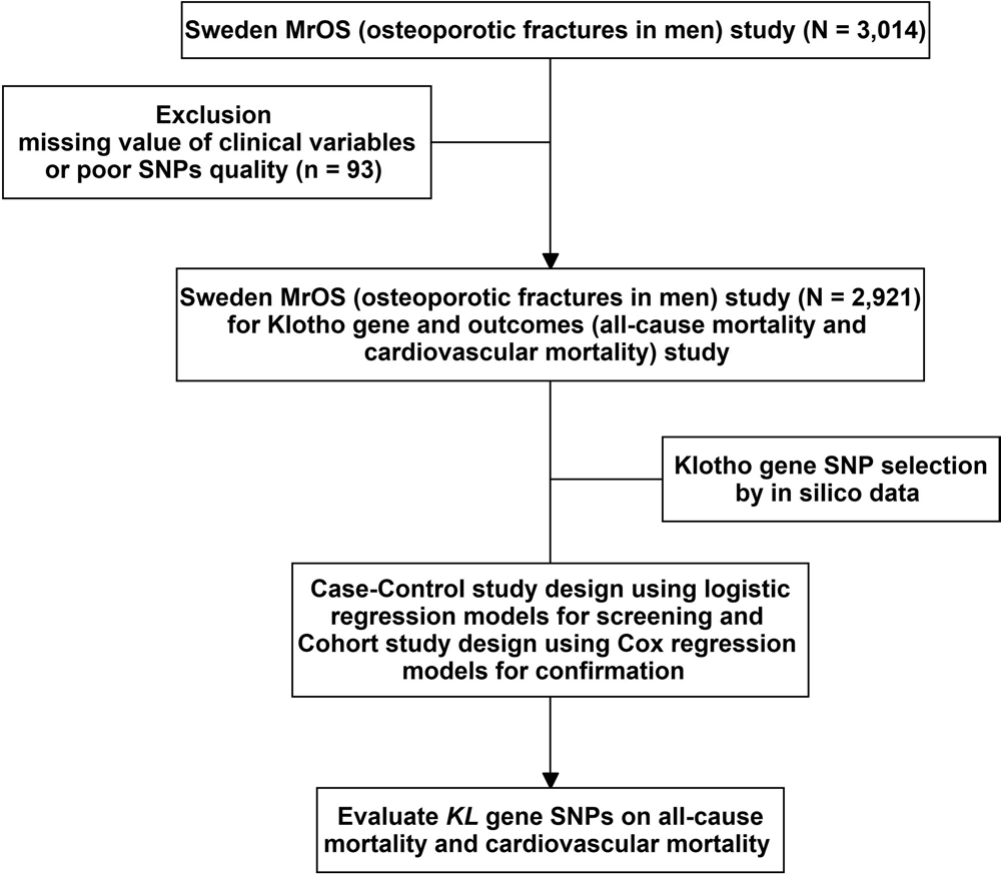
Study design and flow chart.

The present study investigated genotyped SNPs using computational tools to predict damage-associated SNPs in the coding and non-coding region of the human *KL* gene. The selected SNPs in the *KL* gene were then further investigated in the Swedish part of the Osteoporotic Fractures in Men Study (MrOS) cohort (Fig. 1).

### Study population

The MrOS study was a prospective international study of osteoporosis and fracture risk in an elderly male cohort. It included participants in United Status, Sweden, and Hong Kong. We included Sweden MrOS participants aged 69–81 years. The participants (n = 3,014)were randomly selected from national population database, constituting three sub cohorts in Uppsala (n = 999), Göteborg (n = 1,010), and Malmö (n = 1,005). The MrOS Study in Sweden was approved by the ethics committees at Uppsala Universities (Ups 01–057), Göteborg (Gbg M 014–01), and Lund (LU 693-00). The study was performed in accordance with the declaration of Helsinki. Informed consent was obtained from all study participants.

### Data collection, covariates definitions, and mortality assessment

Medical information, such as hypertension, diabetes, myocardial infarction, angina pectoris, stroke, and cancer, was recorded from a standardized questionnaire. Hypertension was defined by baseline self-reported blood pressure-lowering drugs or systolic blood pressure above 140 mmHg, which was measured once after 10 minutes rest in the spine position. Cardiovascular disease (CVD) comorbidity was defined as a history of angina pectoris, myocardial infarction, or stroke. Body mass index (BMI) was estimated as a person’s weight in kilograms, divided by height in meters squared.

Serum samples were collected and stored at −80 °C. Serum concentration of intact FGF23 was analyzed using ELISA (Kainos Laboratories International; Tokyo, Japan)^17^. The estimated glomerular filtration rate (eGFR) cystatin C (Cystatin C Immunoparticles, Dako A/S, Glostrup, Denmark) was calculated using the following formula: eGFR cystatin C = 79.901*(Cyst C [mg/L])^−1.4389^. This proxy for eGFR shows a strong correlation with the iohexol clearance rate (R2 = 0.956)^18,19^.

Mortality was retrieved from Statistics Sweden. Follow-up time was recorded between baseline visit (2001–2004) and the date of death or mortality data collection (March 1, 2008). The cause of death data was recorded by the International Classification of Diseases (ICD) codes based on death certificates from the Swedish Cause of Death Register. CV death was defined by ICD-10 codes I00 to I99 in this study.

### Genotyping of the Klotho gene

DNA was extracted from whole blood using standard methods from 3014 participants. Tagging SNPs covering the *KL* gene and flanking 5’ and 3’ regions were selected using the HaploView 4.2 tagger algorithm^20^. Genotyping was performed using matrix-assisted laser desorption/ionization time-of-flight mass spectrometry (MALDI-TOF MS) on a Sequenom MassARRAY system (Sequenom Inc., Newton, MA) with iPLEX assay. The primers were designed by MassARRAY Assay Design software (Version 3). Polymerase chain reaction (PCR) was performed according to the standard iPLEX methodology. Genotyping quality control was completed by excluding individual samples or SNPs with genotype call rates <95%, SNP assays with poor-quality spectra/cluster plots, and SNPs that significantly deviated from the Hardy-Weinberg equilibrium (p < 0.05) were excluded from the analysis. Genotype data on the SNPs were pairwise calculated regarding linkage disequilibrium and for identification of SNPs with the highest haplotype predictability. Successful genotyping was obtained from 23 SNPs, with an overall call rate of 98.9%. Allele frequencies for these SNPs were calculated and Hardy–Weinberg equilibrium (HWE) was tested for each SNP using chi-square goodness-of-fit statistics in the cohort for all SNP except two (rs1888057 and rs2283368) which were subsequently excluded from further analyses. Haploview 4.2 was accessed to generate haplotype blocks, linkage disequilibrium (LD) values and diagrams, as well as tagging SNPs using the tagger algorithm^20^. The data for chromosome location, minor genotype frequency, SNP function and regulation of the human *KL* gene, and wild or mutated residue of the non-synonymous SNPs (nsSNPs) were used according to the program requirements (Supplementary Tables 1–3).

### Functional analysis Prediction by *in silica* tool

The function of nsSNPs was evaluated by 6 prediction tools, including SIFT (Sorting Intolerant From Tolerant)^21^, Polyphen-2 (Polymorphism Phenotyping v2)^22^, PROVEAN (Protein Variation Effect Analyzer)^23^, SNPs3D^24^, LS-SNP (Large-scale annotation of coding non-synonymous)^25^, and MutPred^26^ (Supplementary Table 4). In addition, computational tools for annotation of genetic variants on both protein-coding and noncoding variants were evaluated by Combined Annotation-Dependent Depletion (CADD)^27^, Deleterious annotation of genetic variants using neural networks (DANN)^28^, FATHMM (Functional Analysis through Hidden Markov Models)^29^, Funseq. 2^30^, Genome-Wide Annotation of VAriants (GWAVA)^31^, PredictSNP2^32^, PhD-SNP (Predictor of Human Deleterious Single Nucleotide Polymorphisms)^33^, and RegulomeDB^34^ (Supplementary Table 4). The prediction tools were selected by using different approaches in order to obtain a classification of the SNPs according to one or more features. Each program’s approach was detailed in the Supplementary Methods.

### Statistical analysis

Patients’ baseline characteristics are reported as the mean ± SD and percentages for continuous and categorical variables, respectively. We performed a two-step procedure. Firstly, logistic regression is used to analyze all of the *KL* gene SNPs and mortality outcomes association as an initial filtering process. Secondly, Cox regression is fitted to those SNPs associated below P-value <0.05 threshold to obtain final results for these SNPs^35^. The association between genotypes for the SNPs and all-cause or CV mortality during follow-up was evaluated by odds ratios (OR) and 95% confidence interval (CI) with homozygotes major allele as reference using additive models. The models were tested: 1) unadjusted and 2) adjusted for age, body mass index, smoking, comorbidities (hypertension, diabetes, coronary artery disease, stroke, cancer), estimated glomerular filtration rate, phosphate, and FGF23 at baseline. In addition, dominant and recessive genetic models were also analyzed. Cox regression model with unadjusted Hazard Ratios (HR) and 95% CI was further analyzed to confirm the finding. Tests for the association and the estimates for the HR were computed with adjustment for age, BMI, and smoking. Since eGFR and FGF23 were strongly correlated with mortality, we further performed subgroup analysis on selected SNPs for mortality stratified by eGFR (≧60 ml/min/1.73 m^2^ and <60 ml/min/1.73 m^2^) and fibroblast growth factor 23 (≧60 pg/ml and <60 pg/ml). Kaplan-Meier curves with log-rank tests were used to examine 6 years of survival according to the *KL* variant. STATA 14 was used for calculations (Stata, College Station, TX, USA). A two-tailed *P* < 0.05 was considered statistically significant.

## Results

### Characteristics of the MrOS Sweden cohort

In MrOS Sweden, 2,921 participants were analyzed after excluding missing value of clinical variables (n = 1) and SNPs quality control (n = 92) (Fig. 1). The baseline characteristics of the MrOS cohort are shown in Table 1. Compared with survivors, participants who died had older age, more smoking habits, more comorbidities (diabetes, CAD, stroke, and cancer), higher FGF23 levels, and lower eGFR levels. The mean follow-up time of the total MrOS cohort was 4.49 ± 1.03 years, 364 deaths occurred and the mortality rate (95% CI) was 26.68 (23.77–29.95)/1,000 person-year in all-cause mortality and 10.93 (9.13–13.09)/1,000 person-year in CV mortality (Table 1).

**Table 1:** Baseline characteristics of participants with and without death in the MrOS cohort.

Demographics	Death (N = 364)	Living patients (N = 2,557)	*P*
Age (years) (mean ± SD)	76.1 ± 3.1	75.3 ± 3.2	<0.001
Body mass index (kg/m²) (mean ± SD)	25.96 ± 3.83	26.47 ± 3.51	0.011
Smokers, N (%)	37 (10.2)	205 (8.0)	0.161
Comorbidities			
Hypertension, N (%)	137 (37.6)	927 (36.3)	0.570
Diabetes, N (%)	54 (14.8)	224 (8.8)	<0.001
Coronary artery disease, N (%)	74 (20.3)	340 (13.3)	<0.001
Stroke, N (%)	37 (10.2)	150 (5.9)	0.002
Cancer, N (%)	88 (24.2)	359 (14.0)	<0.001
Biochemistry			
eGFR (ml/min/1.73 m²) (mean ± SD)	66.76 ± 25.01	72.85 ± 19.70	<0.001
Phosphate (mmol/L) (mean ± SD)	1.09 ± 0.17	1.07 ± 0.16	0.046
FGF23 (pg/ml) (mean ± SD)	53.38 ± 46.51	48.07 ± 33.39	0.009
Deaths N (%)			
All-cause mortality, N (%)	364	—	—
Mortality rate (incidence rate and 95% CI) ^a^	26.68 (23.77–29.95)	—	—
Cardiovascular mortality, N (%)	139 (38.2)	—	—
Mortality rate (incidence rate and 95% CI)^a^	10.93 (9.13–13.09)	—	—
Follow up time (years) (mean ± SD)	2.93 ± 1.36	4.71 ± 0.74	<0.001

Normally distributed continuous variables are presented as mean standard deviation and categorical variables as n (%).

^a^Incidence rate: 1,000 person-year.

eGFR, estimated glomerular filtration rate; FGF23, fibroblast growth factor 23; SD, standard deviation.

### Linkage disequilibrium of SNPs in the Klotho gene calculation

The SNP localizations in the *KL* gene and their degree of linkage (D’) are presented (Supplementary Fig. 1). There are three major haplotype blocks covering 48.6 kb of the gene, with overall linkage disequilibrium over the haplotype blocks exhibiting D’ values above 0.84 (mean max r^2^ in pairwise comparisons 0.983). SNPs rs1888057 and rs2283368 were excluded from further analysis due to deviation from HWE, and rs2249358 and rs541053 were excluded due to call rates less than the 95% predetermined cut-off. SNP rs22227122 was excluded due to a low minor allele frequency of 2.8%. In total, 18 SNPs were taken forward for further downstream analysis. These 18 SNPs included intron variant, transcript variant, missense, and synonymous variant based on Ensembl Variant Effect Predictor (Supplementary Fig. 2). SNPs chromosome position, minor allele frequency (MAF) in different databases, and estimated functional effects are presented in Supplementary Tables 1–3.

### Analysis of SNPs using a combination of bioinformatics tools

Polymorphisms in the *KL* gene were analyzed using computational tools to predict coding and non-coding region SNPs. S*NPs* (rs9536314, rs9527025, rs9527026, rs564481) from the coding region (exon) were selected for further bioinformatic analysis using SIFT, PolyPhen-2, PROVEAN, SNPs3D, LS-SNP, and MutPred. SNP rs9536314 was predicted to be “damaging” in SIFT, “probably damaging” in PolyPhen-2, “deleterious” in PROVEAN, “deleterious” in SNPs3D and MutPred (Supplementary Table 5). SNP rs9527025 was predicted to be “damaging” in SNPs3D, LS-SNP, and MutPred (Supplementary Table 5).

### Klotho SNP and risk of all-cause and cardiovascular mortality

**Figure 2:**
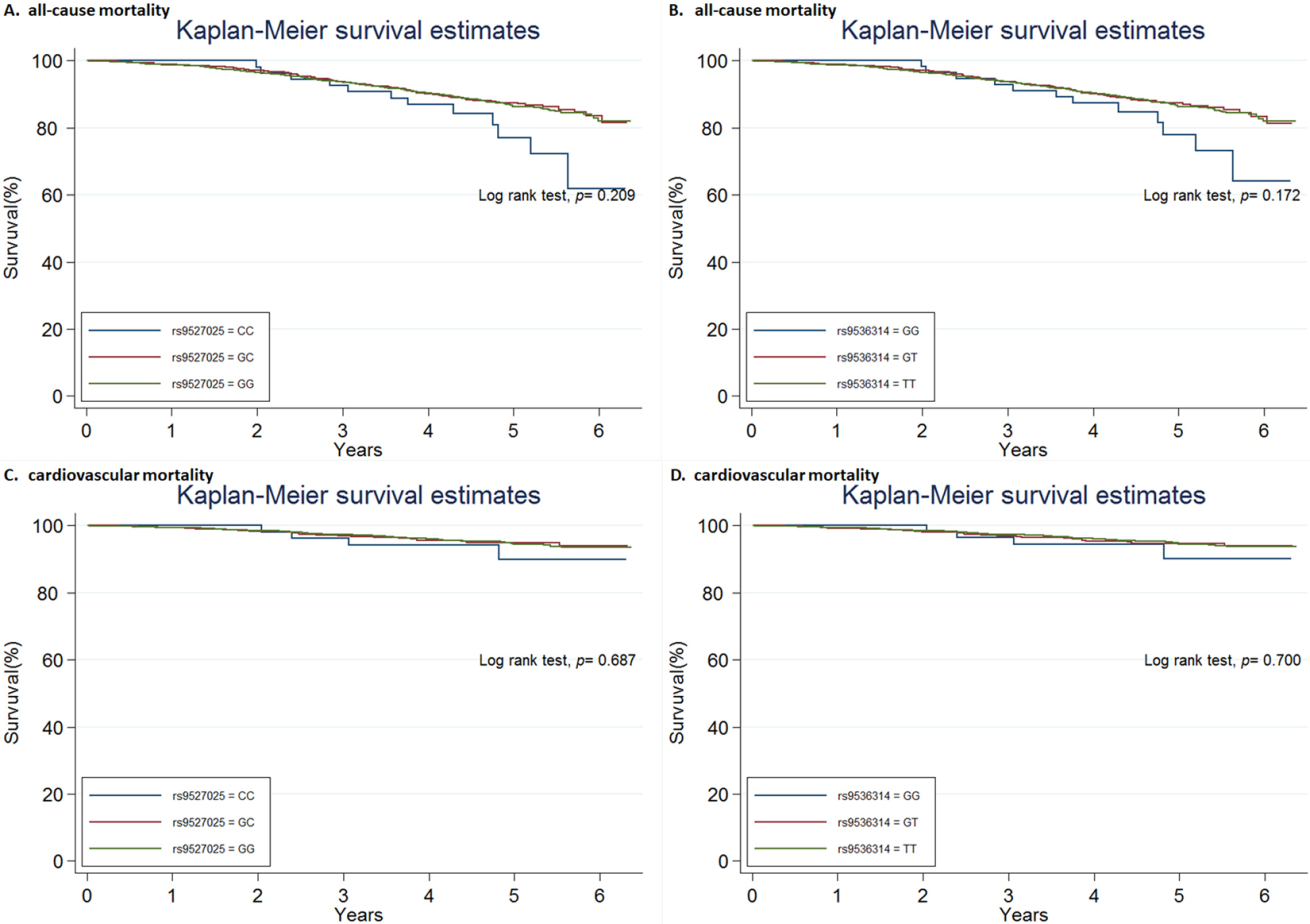
Kaplan-Meier curves of all-cause mortality cardiovascular mortality according to SNP rs9536314 and rs9527025.

**Figure 3:**
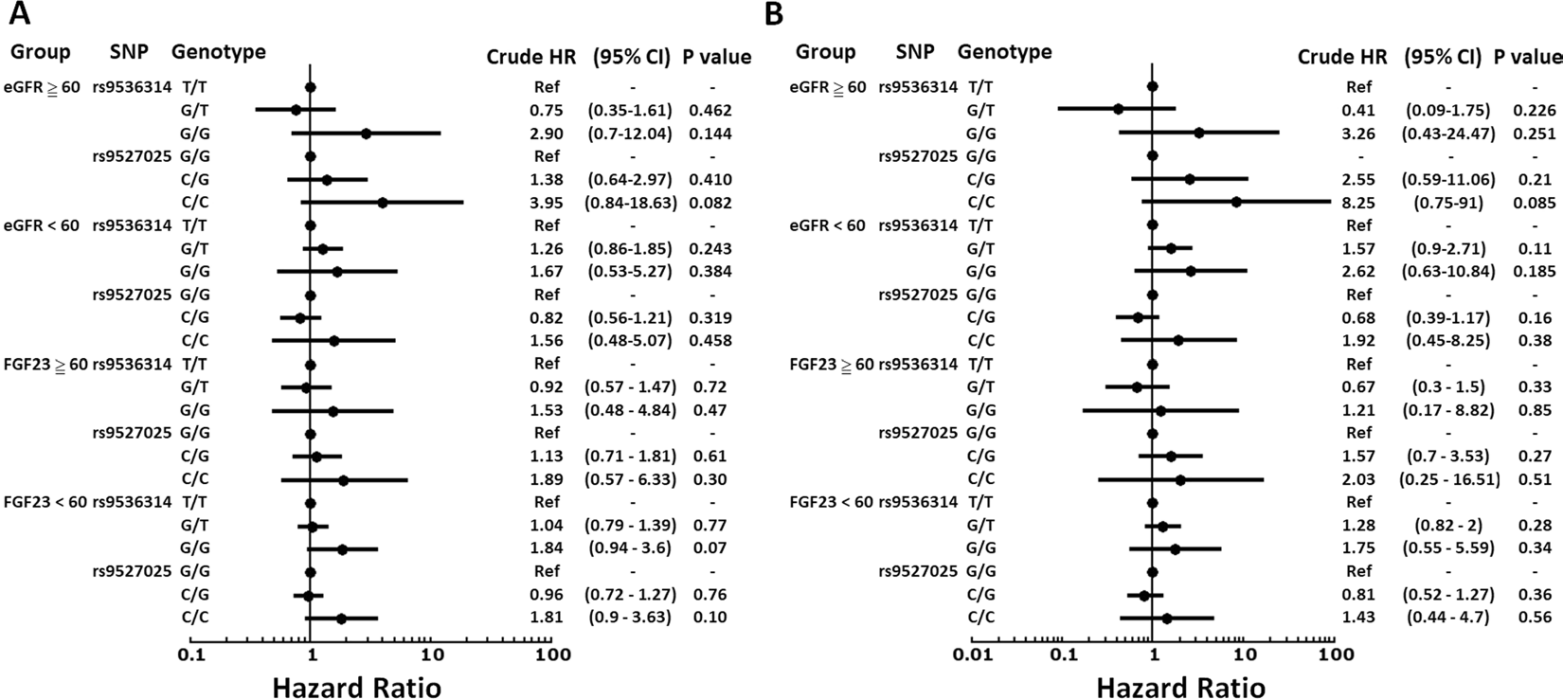
Subgroup analysis of hazard ratio (95% confidence interval) on SNPs rs9536314 and rs9527025 for all-cause mortality (**A**) and cardiovascular mortality (**B**) using univariate Cox regression analysis with major homozygote as reference stratified by estimated glomerular filtration rate (≧60 ml/min/1.73 m^2^ and <60 ml/min/1.73 m^2^) or fibroblast growth factor 23 (≧60 pg/ml and <60 pg/ml).

The total genotype frequency, minor allele frequency, and genotype frequency of death and non-death were shown in Supplementary Tables 1, 6, 7. Using the univariate logistic regression model, there were 3 SNPs associated with increased risk of all-cause mortality in the additive model, including the TT genotype of rs9536282, GG genotype of rs9536314, and CC genotype of rs9527025 (Supplementary Table 8). However, none of the 18 SNPs were statistically significantly associated with the risk of CV mortality (Supplementary Table 8). In adjusted analyses controlling age, body mass index, smoking, comorbidities (hypertension, diabetes, coronary artery disease, stroke, cancer), estimated glomerular filtration rate, phosphate, and FGF23, rs9536282, rs9536314, and rs9527025 genotypes were not associated with all-cause mortality or CV mortality (Supplementary Table 9). Furthermore, in the analyses of dominant and recessive models, rs9527025 and rs9527026 were associated with all-cause mortality under the recessive model (Supplementary Tables 10 and 11). The Kaplan-Meier survival analysis according to genotypes of SNPs rs9536314 and rs9527025 are shown, respectively (Fig. 2). We performed Cox proportional hazards analysis to determine the relationship between rs9536314 and rs9527025 genotype and mortality. No statistical significance was found in the unadjusted and adjusted Cox model (Table 2 and Supplementary Table 12). Subgroup analysis of SNPs rs9536314 and rs9527025 using univariate Cox regression analysis in additive models demonstrated no significant risk difference of all-cause mortality or CV mortality stratified by eGFR or FGF23 (Fig. 3).

**Table 2:** Hazard ratio (95% confidence interval) of SNPs rs9536314 and rs9527025 on all-cause mortality and cardiovascular mortality using univariate Cox regression analysis with major homozygote as reference.

SNP	Genotype	Frequency	All-cause mortality			Cardiovascular mortality		
			Crude HR	(95% CI)	*P* value	Crude HR	(95% CI)	*P* value
rs9536314	T/T	0.733	Ref	—	—	Ref	—	—
	G/T	0.247	0.99	(0.78–1.27)	0.96	1.05	(0.71–1.55)	0.80
	G/G	0.02	1.72	(0.96–3.07)	0.07	1.52	(0.56–4.14)	0.41
rs9527025	G/G	0.729	Ref	—	—	Ref	—	—
	C/G	0.253	1.02	(0.80–1.29)	0.90	0.99	(0.68–1.46)	0.97
	C/C	0.019	1.82	(0.998–3.33)	0.05	1.54	(0.55–4.33)	0.41

HR, hazard ratio; CI, confidence interval.

## Discussion

The SNPs situated in the *KL* gene were evaluated by computational programs and databases that use different methods to predict the damaging of SNPs. The nsSNP rs9536314 and rs9527025 are classified as the most damaging sites. In the clinical study, the risk association of all-cause mortality was found for the TT genotype of rs9536282, GG genotype of rs9536314, and CC genotype of rs9527025 by unadjusted logistic regression analysis using an additive model. However, no statistically significant association was found between polymorphisms in *KL* and mortality risk in the unadjusted Cox regression model or adjusted logistic regression model. The results were similar in the dominant and recessive models. Subgroup analysis of rs9536314 and rs9527025 stratified by eGFR or FGF23 also demonstrated negative results in the MrOS cohort.

In computational data, the nsSNPs are highly significantly responsible for amino acid residue substitutions subsequent in the functional diversity of proteins in humans. Functional variations can have neutral or deleterious effects on protein structure and function^36^. Damaging effects include altering gene regulation, destabilization of protein structure, affecting protein charge, hydrophobicity, geometry, dynamics, stability, translation and inter/intra protein interactions^37–39^. Therefore, it is assumed that nsSNPs will be linked to human disease. However, in order to keep important genetic variants in non-coding regions^40^, we also perform computational tools (CADD^27^, DANN^28^, FATHMM^29^, Funseq. 2^30^, GWAVA^31^, PredictSNP2^32^, PhD-SNP^33^, and RegulomeDB^34^) to discriminate non-coding pathogenic variants from benign variants for complete *KL* gene SNPs evaluation.

We demonstrated a non-significant trend of association between the nsSNPs rs9536314 and rs9527025 on all-cause mortality but not CV mortality. The SNPs of rs9536314 (F352V) and rs9527025 (C370S) in the exon have been reported to be related to CV diseases^9,10^. However, a meta-analysis of rs9536314 (F352V), rs9527025 (C370S) failed to indicate any statistically significant association with the additive genetic model, the dominant genetic model, or the recessive genetic model on CV disease^41^. In addition, the minor allele frequency difference of rs9536314 and rs9527025 between Sweden MrOs data and other databases (1000 Genomes EUR Population, HapMap CEU Population, or Genome Aggregation Database European Population) (Supplementary Table 1) may partly explain the outcomes difference. In our study, although the computational prediction of the “damaging” effect of the *KL-VS* genotype, no statistical significance was found in the study cohort. The contrary results may due to cohorts having different ages, gender, races, and comorbidities. The functional *KL-VS* variant results in amino acid substitutions, altering secretion, catalytic activities, and functionality of the Klotho protein^5,42^. However, a study has also reported that SNPs rs9536314 (F352V) and rs9527025 (C370S) substitutions alter amino acid-related shedding and trafficking but the effects are small and intragenic complementation may further minimize the effects *in vivo*^43^. As for another *KL* gene synonymous variant rs564481 (C1818T) located in the fourth exon, no all-cause or CV mortality association was demonstrated in our study, as compatible with previous studies that no CV disease correlation in this polymorphism^10,41,44^. Although rs564481 is a synonymous variant, it is likely to be functionally relevant, with reports associating it with CV risk factors (blood pressure, glucose metabolism, and lipid levels)^45,46^ and coronary artery disease^47^ in Asian populations. SNP rs577912 has previously been found to be related to higher mortality risk in patients receiving hemodialysis^16^ but negative findings in our clinical result. Interestingly, the computational tool FATHMM predicts rs577912 genotype could be a damaging intron non-coding region but no mortality outcome association was found in this study.

### Study strengths and limitations

Strengths of this study include that the MrOS cohort represents a large sample size of community-dwelling men with nearly complete follow up of surviving cohort participants, and outcome measures are well-validated because of high-quality national registers to obtain information of all-cause and CV death. We perform 14 different computational tools as a bioinformatics approach as well as clinical study to complete the evaluation of *KL* gene polymorphism. Some limitations in this study still need to be addressed. Firstly, self-reported questionnaires were used at baseline visits, so we cannot exclude the possibility of smoking or disease prevalence underestimation. Secondly, the generalizability of the present study is limited to healthy community-dwelling Swedish males. Lastly, the minor allele frequency of rs9536314 homozygotes could be different in elderly and young subjects^5^. This could also affect our study result because of the enrolled elderly population in the MrOS cohort.

## Conclusion

The two potentially damaging SNPs (rs9536314 and rs9527025) in the *KL* gene were not associated with all-cause mortality or CV mortality. Larger scales studies and meta-analysis are needed to confirm the correlation between polymorphisms of the *KL* gene and mortality.

## Supplementary information

Supplementary information
