# Supplementary material for: The association between Single Nucleotide Polymorphisms of Klotho Gene and Mortality in Elderly Men: The MrOS Sweden Study
Source: Sci Rep. 2020 Jun 24;10:10243. doi: 10.1038/s41598-020-66517-5 (PMC7314825; doi:10.1038/s41598-020-66517-5)
Supplement: Supplementary file 1 — Supplementary information [file 41598_2020_66517_MOESM1_ESM.docx]

**The association between Single Nucleotide Polymorphisms of Klotho Gene and Mortality in Elderly Men: The MrOS Sweden Study**

Ping-Hsun Wu^1,5,6,7*^, Per-Anton Westerberg^1*^, Andreas Kindmark^1^, Åsa Tivesten^2^, Magnus K. Karlsson^3^, Dan Mellström^4^, Claes Ohlsson^4^, Bengt Fellström^1^,Torbjörn Linde^1^, Östen Ljunggren^1^

^1^Department of Medical Sciences, Uppsala University, Sweden.

^2^Wallenberg Laboratory for Cardiovascular and Metabolic Research, Institute of Medicine, Sahlgrenska Academy, University of Gothenburg, Gothenburg, Sweden

^3^Clinical and Molecular Osteoporosis Research Unit, Department of Clinical Sciences and Orthopedic Surgery, Lund University, Skåne University Hospital, Sweden.

^4^Center for Bone and Arthritis Research at the Sahlgrenska Academy, Institute of Medicine, Sahlgrenska Academy, University of Gothenburg, Gothenburg, Sweden.

^5^Institute of Clinical Medicine, College of Medicine, Kaohsiung Medical University, Kaohsiung, Taiwan

^6^Faculty of Medicine, College of Medicine, Kaohsiung Medical University, Kaohsiung, Taiwan

^7^Division of Nephrology, Department of Internal Medicine, Kaohsiung Medical University Hospital, Kaohsiung, Taiwan

*Equal contribution

**Corresponding Author**

Östen Ljunggren, MD

Department of Medical Sciences, Uppsala University, Sweden.

University hospital ing 40, 5 tr. SE-751 85 Uppsala, Sweden

Telephone number: +4618-6119080

E-mail address: osten.ljunggren@medsci.uu.se

**Supplementary methods of computational prediction tools**

**Non‑synonymous SNP functional analysis**

The deleterious and damaging effect of Non‑synonymous SNP was predicted using 6 different web-based tools SIFT (Sorting Intolerant from Tolerant), Polyphen-2 (Polymorphism Phenotyping v2), PROVEAN (Protein Variation Effect Analyzer), SNPs3D, LS-SNP (Large-scale annotation of coding non-synonymous), and MutPred. The detail information was demonstrated as follows.

1. SIFT (Sorting Intolerant From Tolerant) tool uses a sequence homology-based on the multiple sequence alignment (MSA) conservation approach to classifying the nsSNPs as tolerated by or damage to the protein. The tolerance index score is a normalized probability that an amino acid substitution is tolerated. Substitutions with a tolerance index less than 0.05 are predicted to be ‘deleterious’ and those with greater than or equal to 0.05 are predicted as ‘tolerated’.^1^

2. Polyphen-2 (Polymorphism Phenotyping v2) is a sequence and structure-based method that determines the structural and functional consequences of nsSNPs. The PolyPhen-2 calculates the posterior probability that an nsSNP is damaging by a Bayesian classifier. Prediction outcomes were classified as probably damaging, possibly damaging or benign according to the score ranging from (0–1). Position-Specific Independent Count (PSIC) score was the probability of the substitution being damaging; “sensitivity” and “specificity” correspond to prediction confidence. The classification of the nsSNPs results in Possibly Damaging and Probably Damaging (PSIC > 0.5) or Benign (PSIC < 0.5). If the score is nearer to 1, nsSNPs will probably be damaging.^2^

3. PROVEAN (Protein Variation Effect Analyzer) measures the damaging effect of variations in protein sequences. The prediction is based on the change, caused by an nsSNP, in the similarity of the sequence to related protein sequences in an MSA. PROVEAN uses a delta alignment score based on the reference and variant versions of the protein sequence with respect to the alignment of homologous sequences. The Cutoff value of PROVEAN is -2.5. amino acid substitutions that have a value greater than cut off value will be considered as deleterious.^3^

4. SNPs3D analyzes the likely impact of nsSNPs on protein function by two methods, one based on the protein structure and stability, stemming from the hypothesis that many disease nsSNPs affect protein function primarily by decreasing protein stability. The program is intended to identify which amino acid substitutions significantly destabilize the folded state. The second model was based on analysis of homology in a sequence of families related to human proteins, through analysis of amino acid conservation at the affected sequence position. A positive SVM score indicates a variant classified as non-deleterious, and a negative score indicates a deleterious variant. The larger the score, the more confident is the classification of the nsSNP, with accuracy significantly higher for scores greater 0.5 or less than-0.5.^4^

5. LS-SNP (Large-scale annotation of coding non-synonymous) uses an SVM trained with rule-based annotation of structure, sequence, and evolution to look for destabilization, proximity to ligands and interfaces and exploits information from OMIM. LS-SNP algorithm included Protein sequences, multiple sequence alignments, protein homology models, predicted domain interfaces, ligand binding sites, hidden Markov models, genes, pathways, and genomic DNA database.^5^

6. MutPred tool was developed to classify an amino acid substitution as deleterious-/disease-associated or neutral, based on three classes of attributes, the evolutionary conservation of the protein sequence, the protein structure and dynamics, and in functional properties, including secondary structure, solvent accessibility, stability, intrinsic disorder, B-factor, transmembrane helix, catalytic residues and others. It determines the changes at the atomic and molecular levels induced by the amino acid substitution. MutPred uses the RF (Random Forest) classifier to provide the g score for the prediction of the probability that the substitution is deleterious, and the *p* score for the indication of the structural and functional properties impacted, for instance, a gain of helical propensity or loss of a phosphorylation site. *G*-value ranges from 0-1 in MutPred results. If the *g*-value is closer to 1 then amino acid substitution shows more effect on protein function.^6^

**Combined coding and Non‑coding SNP functional analysis**

To identify the effect of SNPs in non-coding regions, tools predicting the potential functional effect of SNPs at Transcription factor binding sites (TFBS), Intron/exon border consensus sequences (splice sites), Exonic splicing enhancers (ESEs), and miRNA binding were used. In the present study, we perform 8 computational prediction tools, including CADD (Combined Annotation-Dependent Depletion), DANN (Deleterious annotation of genetic variants using neural networks), FATHMM (Functional Analysis through Hidden Markov Models), Funseq2, GWAVA (Genome-Wide Annotation of VAriants), PredictSNP2, PhD-SNP (Predictor of Human Deleterious Single Nucleotide Polymorphisms), and RegulomeDB. The detail description was shown as below.

1. CADD (Combined Annotation-Dependent Depletion) combines information from 63 different annotations including SIFT, PolyPhen, conservation, predicted effects on regulation, the 'Grantham' score for amino acid differences, using a support vector machine learning to develop its algorithm. It measures deleteriousness by using observed variant frequency as the basis for its calculation. The C score ranges from 1 to 99, with a higher score indicating greater deleteriousness. Values ≧ 10 are predicted to be the 10% most deleterious substitutions, ≧ 20 indicate the 1% most deleterious.^7^

2. DANN (Deleterious annotation of genetic variants using neural networks) uses the same feature set and training data as CADD to train a deep neural network (DNN). DNNs can capture non-linear relationships among features and are better suited than SVMs for problems with a large number of samples and features.^8^

3. FATHMM (Functional Analysis through Hidden Markov Models) combines a conservation-based method using position-specific scoring matrices where a Hidden Markov Model approach and sequence-based conservation features and pathogenicity weights to predict the functional impact of nsSNVs and noncoding variants. Predictions are given as *p*-values range between 0 and 1, with values above 0.5 are predicted to be deleterious, while those below 0.5 are predicted to be neutral or benign. *P*-values close to the extremes (0 or 1) are the highest-confidence predictions that yield the highest accuracy.^9^

4. Funseq2 was developed to annotate and prioritize regulatory somatic mutations in

noncoding regions. The method was built around two main components. A small scale informative data context summarizes data from large-scale genomics such as gene lists, conservation, functional annotations, and network centrality. The variant prioritization pipeline annotates non-coding mutations and prioritizes them against the data context using a weighted scoring scheme accounting for the relative importance of various features. High scores are indicative of high deleteriousness.^10^

5. GWAVA (Genome-Wide Annotation of VAriants) is a tool that aims to predict the functional impact of non-coding genetic variants based on a wide range of annotations of non-coding elements (largely from ENCODE/GENCODE), along with genome-wide properties such as evolutionary conservation and GC-content. GWAVA uses a classifier to discriminate apparently harmless non-coding variants from those that are likely to be involved in the disease. The results include the prediction scores from 3 different versions of the classifier, which are all in the range 0-1 with higher scores indicating variants predicted as more likely to be functional, and the underlying annotations used to compute these scores.^11^

6. PredictSNP2 is a unified platform for evaluating SNP effects by exploiting the different characteristics of variants. It is used to develop category-optimal decision thresholds and to evaluate six tools for variant prioritization: CADD, DANN, FATHMM, FitCons, FunSeq2, and GWAVA. This evaluation revealed some important advantages of the category-based approach. The results obtained with the five best-performing tools were then combined into a consensus score.^12^

7. PhD-SNP (Predictor of Human Deleterious Single Nucleotide Polymorphisms) is an SVM-based classifier that uses protein sequence information to predict whether an nsSNP is disease-associated, based on a million amino acid polymorphism datasets

using supervised training. The output is obtained from the frequencies of the wild and mutant residues, the number of aligned sequences, and the reliability index score calculated for the position involved, and provides a prediction of disease or neutral polymorphism.^13^

8. RegulomeDB is a database providing a functional annotation of SNPs with known and predicted regulatory elements in the intergenic regions of the human genome. The database includes up-to-date high-quality datasets from Encyclopedia of DNA Elements transcription factor, chromatin immunoprecipitation sequencing (ChIP-seq), histone ChIP-seq, Formaldehyde-Assisted Isolation of Regulatory Elements, DNase I hypersensitive site data and other sources like a large collection of Expression quantitative trait loci, dsQTL, ChIP-exo data to identify putative regulatory variants. Variants can be classified into one of four RegulomeDB categories with scores ranging from 1 to 6 indicating putative functions.^14^

**Supplementary Figure 1.** Localization of the SNPs on *KL* and their linkage using Haploview to generate haplotype blocks


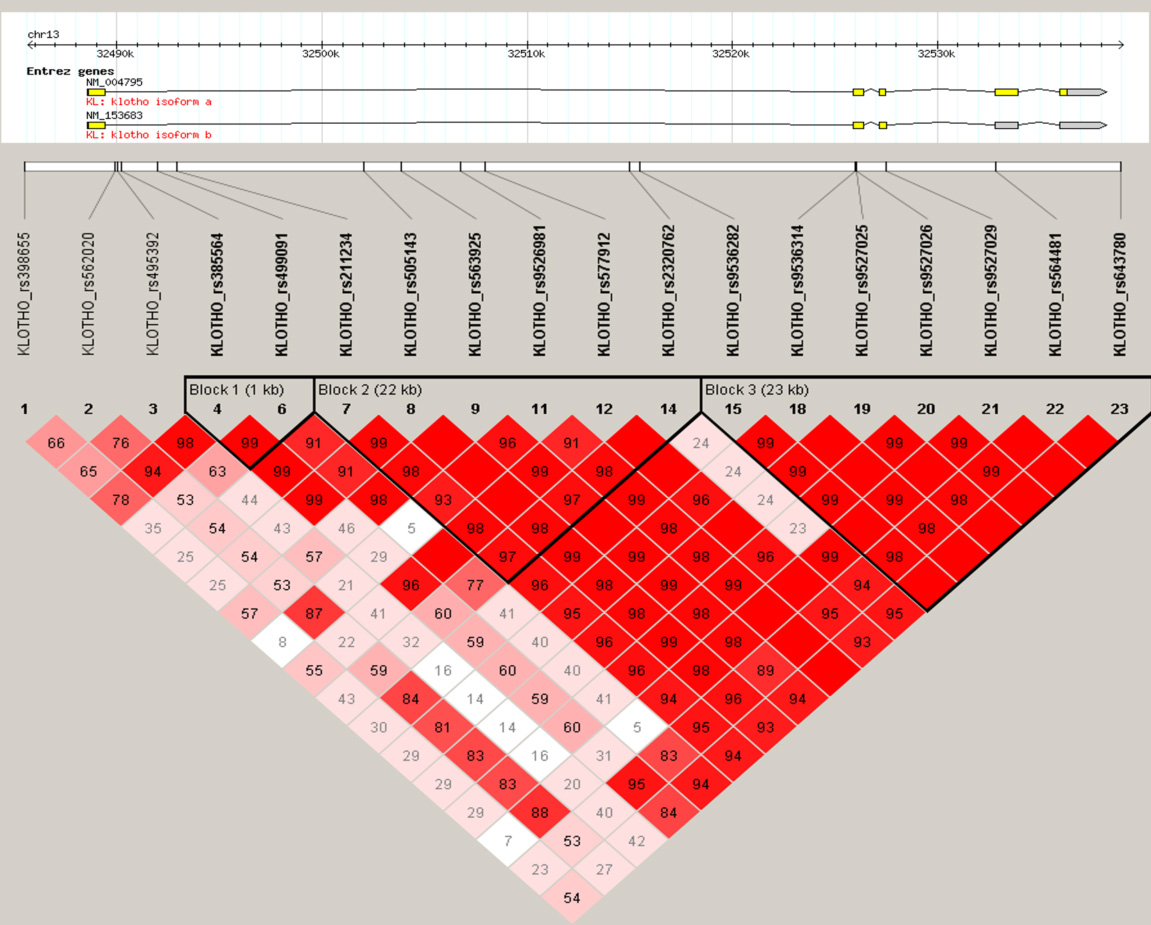


**Supplementary Figure 2.** The enrolled SNPs information based on Ensembl Variant Effect Predictor


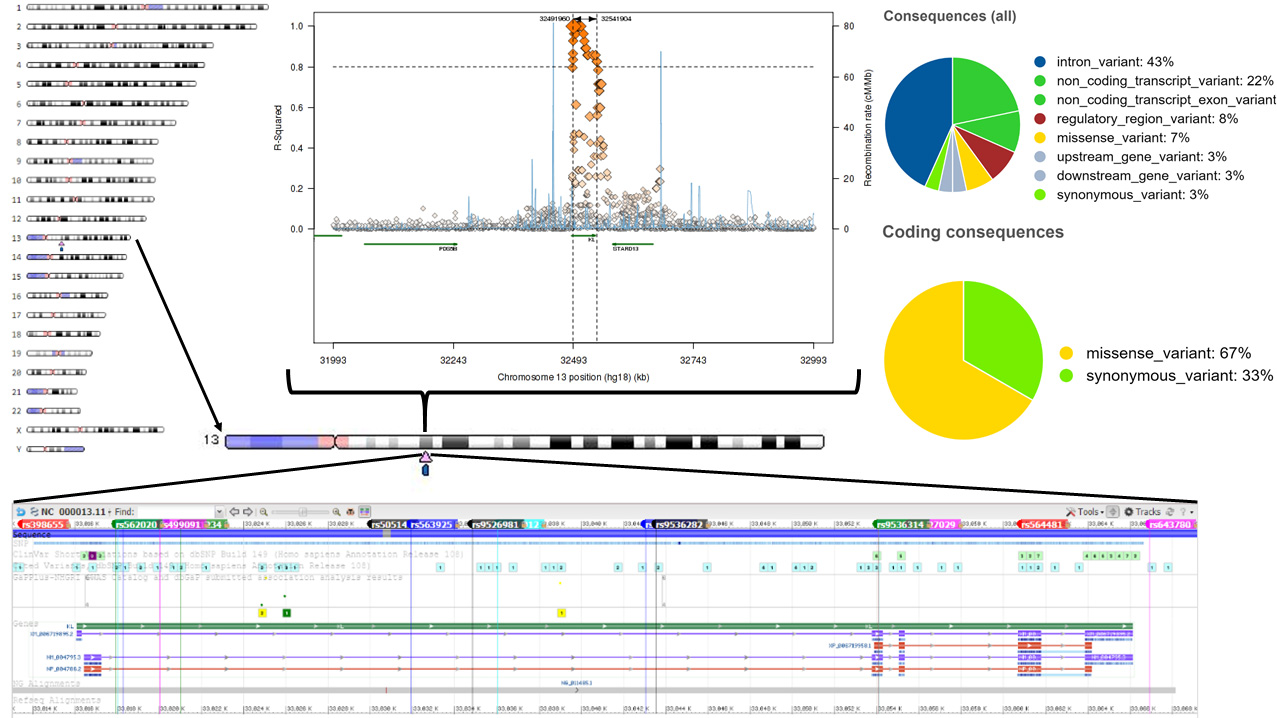


**Supplementary Table 1.** Klotho gene SNP data and minor allele frequency

| SNP | Chromosome Position (GRCh38) | Chromosome Position (GRCh37) | Major allele | Minor allele | MAF (1000 Genomes EUR Population) | MAF(HapMap CEU Population) | MAF (gnomAD European) | Validation* | MAF in our study** |
| --- | --- | --- | --- | --- | --- | --- | --- | --- | --- |
| rs398655 | 33013514 | 33587652 | A | C | 0.441 | 0.407 | 0.440 | Y | 0.448 |
| rs562020 | 33017932 | 33592070 | G | A | 0.346 | 0.333 | 0.338 | Y | 0.327 |
| rs495392 | 33018055 | 33592193 | C | A | 0.261 | 0.270 | 0.280 | Y | 0.264 |
| rs385564 | 33018271 | 33592409 | C | G | 0.349 | 0.342 | 0.313 | Y | 0.307 |
| rs499091 | 33020018 | 33594156 | A | G | 0.38 | 0.398 | 0.406 | Y | 0.409 |
| rs211234 | 33020990 | 33595128 | G | A | 0.351 | - | 0.387 | Y | 0.393 |
| rs505143 | 33030047 | 33604185 | G | A | 0.351 | 0.286 | 0.387 | Y | 0.396 |
| rs563925 | 33031920 | 33606057 | C | A | 0.328 | 0.366 | - | Y | 0.315 |
| rs9526981 | 33034820 | 33608957 | T | G | 0.096 | 0.133 | 0.078 | N | 0.079 |
| rs577912 | 33036014 | 33610151 | G | T | 0.123 | 0.146 | 0.140 | Y | 0.143 |
| rs2320762 | 33043037 | 33617174 | T | G | 0.43 | 0.434 | 0.386 | Y | 0.388 |
| rs9536282 | 33043525 | 33617662 | C | T | 0.198 | 0.174 | 0.163 | Y | 0.144 |
| rs9536314 | 33054001 | 33628138 | T | G | 0.194 | 0.175 | 0.164 | Y | 0.143 |
| rs9527025 | 33054056 | 33628193 | G | C | 0.194 | 0.175 | 0.164 | Y | 0.145 |
| rs9527026 | 33054102 | 33628239 | G | A | 0.194 | 0.168 | 0.162 | Y | 0.144 |
| rs9527029 | 33055520 | 33629657 | C | T | 0.101 | 0.038 | 0.081 | Y | 0.078 |
| rs564481 | 33060846 | 33634983 | C | T | 0.362 | - | 0.393 | Y | 0.403 |
| rs643780 | 33066938 | 33641075 | G | A | 0.123 | 0.146 | 0.141 | Y | 0.145 |

*Validation status description based on (1) Validated by multiple, independent submissions to the ref SNP cluster (2) Validated by frequency or genotype data: minor alleles observed in at least two chromosomes. (3) Validated by submitter confirmation (4) All alleles have been observed in at least two chromosomes apiece (5) Genotyped by HapMap project (6) SNP has been sequenced in 1000Genome project.

**The minor allele frequency in our study was calculated based on 2921 male participants in Sweden MrOs study

Abbreviations: MAF, minor allele frequency; SNP, single nucleotide polymorphism; gnomAD, Genome Aggregation Database

**Supplementary Table 2.** Klotho gene SNP functional effects from rSNPBase database

| SNP | regulatory-SNP (rSNP) | LD-proxy of rSNP(r^2^>0.8) | Proximal regulation | Distal regulation | miRNA regulation | RNA binding protein mediated regulation | Expression Quantitative trait loci (eQTL) |
| --- | --- | --- | --- | --- | --- | --- | --- |
| rs398655 | Y | Y | Y | N | N | N | N |
| rs562020 | Y | N | N | N | N | Y | Y |
| rs495392 | Y | Y | N | N | N | Y | Y |
| rs385564 | Y | Y | N | N | N | Y | Y |
| rs499091 | Y | Y | N | N | N | Y | N |
| rs211234 | Y | Y | N | N | N | Y | N |
| rs505143 | Y | Y | N | N | N | Y | N |
| rs563925 | Y | Y | N | N | N | Y | N |
| rs9526981 | Y | Y | N | N | N | Y | N |
| rs577912 | Y | Y | N | N | N | Y | Y |
| rs2320762 | Y | Y | N | N | N | Y | Y |
| rs9536282 | Y | Y | N | N | N | Y | Y |
| rs9536314 | Y | Y | N | N | N | Y | Y |
| rs9527025 | Y | Y | N | N | N | Y | N |
| rs9527026 | Y | Y | N | N | N | Y | Y |
| rs9527029 | Y | Y | N | Y | N | Y | N |
| rs564481 | Y | Y | Y | N | N | Y | N |
| rs643780 | N | Y | N | N | N | N | Y |

**Supplementary Table 3.** Klotho SNPs and the encoded amino acids

| SNP | Gene region function | TF binding sites | CDS position | mRNA association No. | Protein association No. | Protein ID | UniProt ID | synonymous/non-synonymous coding SNPs | Protein position | Amino acids | OMIM ID | Mutation related disease |
| --- | --- | --- | --- | --- | --- | --- | --- | --- | --- | --- | --- | --- |
| rs398655 | upstream gene variant | Y | - | - | - | - | - | - | - | - | - | - |
| rs562020 | intron variant | - | - | - | - | - | - | - | - | - | - | - |
| rs495392 | intron variant | - | - | - | - | - | - | - | - | - | - | - |
| rs385564 | intron variant | - | - | - | - | - | - | - | - | - | - | - |
| rs499091 | intron variant | - | - | - | - | - | - | - | - | - | - | - |
| rs211234 | intron variant | - | - | - | - | - | - | - | - | - | - | - |
| rs505143 | intron variant | - | - | - | - | - | - | - | - | - | - | - |
| rs563925 | intron variant | - | - | - | - | - | - | - | - | - | - | - |
| rs9526981 | intron variant | - | - | - | - | - | - | - | - | - | - | - |
| rs577912 | intron variant | - | - | - | - | - | - | - | - | - | - | - |
| rs2320762 | intron variant | - | - | - | - | - | - | - | - | - | - | - |
| rs9536282 | intron variant | - | - | - | - | - | - | - | - | - | - | - |
| rs9536314 | exon variant | - | 1054 | NM_004795 | NP_004786 | ENSP00000369442 | Q9UEF7 | missense variant | 352 | Phe/Val | 604824 | CAD |
| rs9527025 | exon variant | - | 1109 | NM_004795 | NP_004786 | ENSP00000369442 | Q9UEF7 | missense variant | 370 | Cys/Ser | 604824 | CAD |
| rs9527026 | exon variant | - | 1155 | NM_004795 | NP_004786 | ENSP00000369442 | Q9UEF7 | synonymous variant | 385 | Lys/Lys | 604824 | CAD |
| rs9527029 | intron variant | - | - | - | - | - | - | - | - | - | - | - |
| rs564481 | exon variant | - | 1767 | NM_004795 | NP_004786 | ENSP00000369442 | Q9UEF7 | synonymous variant | 589 | His/His | 604824 | CAD |
| rs643780 | downstream gene variant | - | - | - | - | - | - | - | - | - | - | - |

TF, Transcription factor; CDS, coding DNA sequence (CDS) position; CAD, Coronary artery disease

**Supplementary Table 4.** Prediction tools used in the analysis

| Prediction tool | URL | Type | Reference |
| --- | --- | --- | --- |
| SIFT | <http://sift.jcvi.org/> | evolutionary conservation-based | ^1^ |
| PolyPhen-2 | <http://genetics.bwh.harvard.edu/pph2/index.shtml> | evolutionary conservation and structure-based | ^2^ |
| PROVEAN | <http://provean.jcvi.org/> | evolutionary conservation-based | ^3^ |
| SNPs3D | <http://snps3d.org/> | machine learning method (SVM) | ^4^ |
| LS-SNP | <http://modbase.compbio.ucsf.edu/LS-SNP/> | machine learning method (SVM) | ^5^ |
| MutPred | <http://mutpred.mutdb.org/> | evolutionary conservation and structure-based | ^6^ |
| CADD | <http://cadd.gs.washington.edu/> | evolutionary conservation and machine learning method (SVM) | ^7^ |
| DANN | <https://cbcl.ics.uci.edu/public_data/DANN/> | evolutionary conservation and machine learning method (deep learning) | ^8^ |
| FATHMM | <http://fathmm.biocompute.org.uk/> | machine learning method (Hidden Markov Models) | ^9^ |
| FunSeq2 | <http://funseq2.gersteinlab.org/> | evolutionary conservation | ^10^ |
| GWAVA | <https://www.sanger.ac.uk/sanger/StatGen_Gwava> | machine-learning method | ^11^ |
| PredictSNP^2^ | <https://loschmidt.chemi.muni.cz/predictsnp2/> | category-based approach of CADD, DANN, FATHMM, FitCons, FunSeq2 and GWAVA. | ^12^ |
| PhD-SNPg | <http://snps.biofold.org/phd-snpg/> | machine-learning method based on CADD and FATHMM | ^13^ |
| Regulome DB | <http://www.regulomedb.org/> | evolutionary conservation | ^14^ |

**Supplementary table 5.** Klotho SNP coding variants and noncoding variants annotation

| SNP | SIFT | PolyPhen-2 | PROVEAN | SNPs3D | LS-SNP | MutPred | CADD | DANN | FATHMM | FunSeq2 | GWAVA | PredictSNP2 | PhD-SNP^g^ |
| --- | --- | --- | --- | --- | --- | --- | --- | --- | --- | --- | --- | --- | --- |
| rs398655 | - | - | - | - | - | - | neutral | neutral | neutral | deleterious | neutral | neutral | benign |
| rs562020 | - | - | - | - | - | - | neutral | neutral | neutral | deleterious | neutral | neutral | benign |
| rs495392 | - | - | - | - | - | - | neutral | neutral | neutral | neutral | neutral | neutral | benign |
| rs385564 | - | - | - | - | - | - | neutral | neutral | neutral | deleterious | neutral | neutral | benign |
| rs499091 | - | - | - | - | - | - | neutral | neutral | neutral | neutral | neutral | neutral | benign |
| rs211234 | - | - | - | - | - | - | neutral | deleterious | neutral | neutral | neutral | neutral | benign |
| rs505143 | - | - | - | - | - | - | neutral | neutral | neutral | neutral | neutral | neutral | benign |
| rs563925 | - | - | - | - | - | - | neutral | neutral | neutral | neutral | neutral | neutral | benign |
| rs9526981 | - | - | - | - | - | - | neutral | neutral | neutral | neutral | neutral | neutral | pathogenic |
| rs577912 | - | - | - | - | - | - | neutral | neutral | deleterious | neutral | neutral | neutral | benign |
| rs2320762 | - | - | - | - | - | - | neutral | neutral | neutral | neutral | neutral | neutral | benign |
| rs9536282 | - | - | - | - | - | - | neutral | neutral | neutral | neutral | neutral | neutral | benign |
| rs9536314 | damaging | probably damaging | Deleterious | damaging | - | damaging | neutral | neutral | deleterious | neutral | deleterious | deleterious | pathogenic |
| rs9527025 | tolerated | neutral | neutral | damaging | destabilizing | damaging | deleterious | neutral | neutral | neutral | deleterious | neutral | benign |
| rs9527026 | tolerated | - | neutral | - | - | damaging | neutral | neutral | deleterious | neutral | neutral | neutral | benign |
| rs9527029 | - | - | - | - | - | - | neutral | neutral | neutral | neutral | neutral | neutral | benign |
| rs564481 | tolerated | neutral | - | - | - | damaging | neutral | neutral | neutral | neutral | neutral | neutral | benign |
| rs643780 | - | - | - | - | - | - | neutral | neutral | neutral | neutral | neutral | neutral | benign |

SIFT, Sorting Intolerant From Tolerant; PolyPhen-2, Polymorphism Phenotyping v2; PROVEAN, Protein Variation Effect Analyzer; LS-SNP, Large-Scale Annotation of Coding non-synonymous; CADD, Combined Annotation Dependent Depletion; DANN, Deleterious Annotation of genetic variants using Neural Networks, FATHMM, Functional Analysis Through Hidden Markov Models; GWAVA, Genome-Wide Annotation of Variants

Klotho SNP rs9536314, rs9527025, rs9527026, and rs564481 are coding variants. Other SNPs are noncoding variants.

**Supplementary Table 6.** Klotho gene genotype frequencies

| Single-nucleotide polymorphism | Genotype | N (%) |
| --- | --- | --- |
| rs398655 | A/A | 864 ( 30.3 ) |
|  | A/C | 1,423 ( 49.9 ) |
|  | C/C | 565 ( 19.8 ) |
| rs562020 | G/G | 1,278 ( 45.0 ) |
|  | G/A | 1,266 ( 44.6 ) |
|  | A/A | 296 ( 10.4 ) |
| rs495392 | C/C | 1,535 ( 54.1 ) |
|  | C/A | 1,103 ( 38.9 ) |
|  | A/A | 198 ( 7.0 ) |
| rs385564 | C/C | 1,359 ( 48.1 ) |
|  | C/G | 1,200 ( 42.5 ) |
|  | G/G | 267 ( 9.4 ) |
| rs499091 | A/A | 991 ( 34.9 ) |
|  | A/G | 1,370 ( 48.3 ) |
|  | G/G | 476 ( 16.8 ) |
| rs211234 | G/G | 1,056 ( 37.0 ) |
|  | G/A | 1,350 ( 47.3 ) |
|  | A/A | 446 ( 15.6 ) |
| rs505143 | G/G | 1,048 ( 36.7 ) |
|  | G/A | 1,350 ( 47.3 ) |
|  | A/A | 454 ( 15.9 ) |
| rs563925 | C/C | 1,341 ( 47.6 ) |
|  | C/A | 1,181 ( 41.9 ) |
|  | A/A | 298 ( 10.6 ) |
| rs9526981 | T/T | 2,431 ( 85.1 ) |
|  | T/G | 405 ( 14.2 ) |
|  | G/G | 22 ( 0.8 ) |
| rs577912 | G/G | 2,072 ( 73.1 ) |
|  | G/T | 717 ( 25.3 ) |
|  | T/T | 46 ( 1.6 ) |
| rs2320762 | T/T | 1,085 ( 38.1 ) |
|  | T/G | 1,316 ( 46.2 ) |
|  | G/G | 446 ( 15.7 ) |
| rs9536282 | C/C | 2,090 ( 73.1 ) |
|  | C/T | 713 ( 24.9 ) |
|  | T/T | 56 ( 2.0 ) |
| rs9536314 | T/T | 2,101 ( 73.3 ) |
|  | T/G | 709 ( 24.7 ) |
|  | G/G | 56 ( 2.0 ) |
| rs9527025 | G/G | 2,080 ( 72.9 ) |
|  | G/C | 721 ( 25.3 ) |
|  | C/C | 54 ( 1.9 ) |
| rs9527026 | G/G | 2,080 ( 73.2 ) |
|  | G/A | 710 ( 24.9 ) |
|  | A/A | 56 ( 2.0 ) |
| rs9527029 | C/C | 2,441 ( 85.2 ) |
|  | C/T | 404 ( 14.1 ) |
|  | T/T | 21 ( 0.7 ) |
| rs564481 | C/C | 1,028 ( 35.9 ) |
|  | C/T | 1,365 ( 47.6 ) |
|  | T/T | 474 ( 16.5 ) |
| rs643780 | G/G | 2,088 ( 72.8 ) |
|  | G/A | 729 ( 25.4 ) |
|  | A/A | 53 ( 1.8 ) |

**Supplementary Table 7**. Genotype frequency of death and non-death patients

| Single-nucleotide polymorphism | Genotype | Non-Deaths (%) | Deaths (%) | *P*-value |
| --- | --- | --- | --- | --- |
| rs398655 | C/C | 496 (19.9) | 69 (19.5) | 0.841 |
|  | C/A | 1250 (50.0) | 173 (48.9) |  |
|  | A/A | 752 (30.1) | 112 (31.6) |  |
| rs562020 | G/G | 1124 (45.1) | 154 (44.0) | 0.554 |
|  | G/A | 1102 (44.3) | 164 (46.9) |  |
|  | A/A | 264 (10.6) | 32 (9.1) |  |
| rs495392 | C/C | 1358 (54.5) | 177 (51.3) | 0.298 |
|  | C/A | 956 (38.4) | 147 (42.6) |  |
|  | A/A | 177 (7.1) | 21 (6.1) |  |
| rs385564 | C/C | 1192 (48.1) | 167 (48.0) | 0.820 |
|  | C/G | 1055 (42.6) | 145 (41.7) |  |
|  | G/G | 231 (9.3) | 36 (10.3) |  |
| rs499091 | A/A | 877 (35.3) | 114 (32.6) | 0.392 |
|  | A/G | 1189 (47.8) | 181 (51.7) |  |
|  | G/G | 421 (16.9) | 55 (15.7) |  |
| rs211234 | G/G | 931 (37.3) | 125 (35.3) | 0.692 |
|  | G/A | 1175 (47.0) | 175 (49.4) |  |
|  | A/A | 392 (15.7) | 54 (15.3) |  |
| rs505143 | G/G | 929 (37.2) | 119 (33.4) | 0.319 |
|  | G/A | 1169 (46.8) | 181 (50.8) |  |
|  | A/A | 398 (15.9) | 56 (15.7) |  |
| rs563925 | C/C | 1177 (47.6) | 164 (47.3) | 0.297 |
|  | C/A | 1027 (41.5) | 154 (44.4) |  |
|  | A/A | 269 (10.9) | 29 (8.4) |  |
| rs9526981 | T/T | 2126 (85.0) | 305 (85.4) | 0.667 |
|  | T/G | 357 (14.3) | 48 (13.4) |  |
|  | G/G | 18 (0.7) | 4 (1.1) |  |
| rs577912 | G/G | 1808 (72.8) | 264 (75.2) | 0.535 |
|  | G/T | 634 (25.5) | 83 (23.6) |  |
|  | T/T | 42 (1.7) | 4 (1.1) |  |
| rs2320762 | T/T | 949 (38.1) | 136 (38.4) | 0.487 |
|  | T/G | 1146 (46.0) | 170 (48.0) |  |
|  | G/G | 398 (16.0) | 48 (13.6) |  |
| rs9536282 | C/C | 1832 (73.2) | 258 (72.3) | 0.123 |
|  | C/T | 626 (25.0) | 87 (24.4) |  |
|  | T/T | 44 (1.8) | 12 (3.4) |  |
| rs9536314 | T/T | 1845 (73.5) | 256 (71.9) | 0.117 |
|  | T/G | 621 (24.7) | 88 (24.7) |  |
|  | G/G | 44 (1.8) | 12 (3.4) |  |
| rs9527025 | G/G | 1827 (73.1) | 253 (71.5) | 0.086 |
|  | G/C | 632 (25.3) | 89 (25.1) |  |
|  | C/C | 42 (1.7) | 12 (3.4) |  |
| rs9527026 | G/G | 1832 (73.3) | 256 (71.9) | 0.122 |
|  | G/A | 622 (24.9) | 88 (24.7) |  |
|  | A/A | 44 (1.8) | 12 (3.4) |  |
| rs9527029 | C/C | 2137 (85.1) | 304 (85.6) | 0.752 |
|  | C/T | 357 (14.2) | 47 (13.2) |  |
|  | T/T | 17 (0.7) | 4 (1.1) |  |
| rs564481 | C/C | 908 (36.1) | 120 (33.8) | 0.688 |
|  | C/T | 1191 (47.4) | 174 (49.0) |  |
|  | T/T | 413 (16.4) | 61 (17.2) |  |
| rs643780 | G/G | 1815 (72.3) | 273 (76.3) | 0.280 |
|  | G/A | 650 (25.9) | 79 (22.1) |  |
|  | A/A | 47 (1.9) | 6 (1.7) |  |

**Supplementary Table 8.** Odds ratio (95% confidence interval) of the genotype for *KL* SNPs on all-cause mortality and cardiovascular mortality using univariate logistic regression analysis with major homozygote as reference

| SNP |  | All-cause mortality | | | Cardiovascular mortality | | |
| --- | --- | --- | --- | --- | --- | --- | --- |
|  | Allele | Crude OR | (95% CI) | *P* value | Crude OR | (95% CI) | *P* value |
| rs398655 | C/C | Ref | - | - | Ref |  | - |
|  | C/A | 1.01 | (0.75 - 1.35) | 0.97 | 1.14 | (0.74 - 1.76) | 0.56 |
|  | A/A | 1.08 | (0.83 - 1.39) | 0.57 | 0.90 | (0.60 - 1.35) | 0.62 |
| rs562020 | G/G | Ref | - | - | Ref |  | - |
|  | G/A | 1.09 | (0.86 - 1.37) | 0.49 | 1.30 | (0.91 - 1.87) | 0.15 |
|  | A/A | 0.88 | (0.59 - 1.32) | 0.55 | 1.02 | (0.55 - 1.90) | 0.95 |
| rs495392 | C/C | Ref | - | - | Ref |  | - |
|  | C/A | 0.85 | (0.67 - 1.07) | 0.17 | 0.76 | (0.53 - 1.08) | 0.12 |
|  | A/A | 0.77 | (0.48 - 1.25) | 0.29 | 0.81 | (0.40 - 1.67) | 0.57 |
| rs385564 | C/C | Ref | - | - | Ref | - | - |
|  | G/C | 1.11 | (0.76 - 1.64) | 0.59 | 0.79 | (0.40 - 1.55) | 0.49 |
|  | G/G | 0.98 | (0.77 - 1.24) | 0.87 | 1.10 | (0.77 - 1.58) | 0.59 |
| rs499091 | A/A | Ref | - | - | Ref | - | - |
|  | G/A | 1.01 | (0.71 - 1.42) | 0.98 | 0.99 | (0.58 - 1.71) | 0.97 |
|  | G/G | 1.17 | (0.91 - 1.50) | 0.22 | 1.35 | (0.92 - 1.98) | 0.13 |
| rs211234 | G/G | Ref | - | - | Ref | - | - |
|  | A/G | 0.90 | (0.71 - 1.15) | 0.41 | 0.84 | (0.58 - 1.22) | 0.36 |
|  | A/A | 0.92 | (0.67 - 1.28) | 0.64 | 0.67 | (0.39 - 1.17) | 0.16 |
| rs505143 | G/G | Ref | - | - | Ref | - | - |
|  | A/G | 1.21 | (0.94 - 1.55) | 0.13 | 1.28 | (0.88 - 1.87) | 0.20 |
|  | A/A | 1.10 | (0.78 - 1.54) | 0.59 | 0.90 | (0.52 - 1.57) | 0.71 |
| rs563925 | C/C | Ref | - | - | Ref | - | - |
|  | A/C | 0.93 | (0.73 - 1.18) | 0.54 | 0.97 | (0.67 - 1.39) | 0.86 |
|  | A/A | 0.72 | (0.47 - 1.09) | 0.12 | 0.87 | (0.47 - 1.60) | 0.65 |
| rs9526981 | T/T | Ref | - | - | Ref | - | - |
|  | G/T | 0.94 | (0.68 - 1.30) | 0.70 | 1.03 | (0.63 - 1.67) | 0.91 |
|  | G/G | 1.55 | (0.52 - 4.61) | 0.43 | 1.98 | (0.46 - 8.56) | 0.36 |
| rs577912 | G/G | Ref | - | - | Ref | - | - |
|  | T/G | 0.90 | (0.69 - 1.17) | 0.42 | 0.90 | (0.60 - 1.35) | 0.62 |
|  | T/T | 0.65 | (0.23 - 1.83) | 0.42 | 1.35 | (0.41 - 4.42) | 0.62 |
| rs2320762 | T/T | Ref | - | - | Ref | - | - |
|  | G/T | 1.04 | (0.81 - 1.32) | 0.78 | 1.05 | (0.72 - 1.53) | 0.79 |
|  | G/G | 0.84 | (0.59 - 1.19) | 0.33 | 1.00 | (0.60 - 1.69) | 0.99 |
| rs9536282 | C/C | Ref | - | - | Ref |  | - |
|  | C/T | 0.99 | (0.76 - 1.28) | 0.92 | 1.03 | (0.69 - 1.52) | 0.89 |
|  | T/T | 1.94 | (1.01 - 3.72) | 0.047* | 1.53 | (0.54 - 4.32) | 0.42 |
| rs9536314 | T/T | Ref | - | - | Ref | - | - |
|  | G/T | 1.02 | (0.79 - 1.32) | 0.87 | 1.07 | (0.72 - 1.59) | 0.73 |
|  | G/G | 1.97 | (1.02 - 3.77) | 0.04* | 1.59 | (0.56 - 4.48) | 0.38 |
| rs9527025 | G/G | Ref | - | - | Ref | - | - |
|  | C/G | 0.98 | (0.76 - 1.27) | 0.90 | 0.97 | (0.65 - 1.44) | 0.88 |
|  | C/C | 2.03 | (1.03 - 4.00) | 0.04* | 1.57 | (0.54 - 4.59) | 0.41 |
| rs9527026 | G/G | Ref | - | - | Ref | - | - |
|  | A/G | 0.99 | (0.76 - 1.28) | 0.93 | 0.97 | (0.65 - 1.44) | 0.88 |
|  | A/A | 1.93 | (0.98 - 3.79) | 0.06 | 1.48 | (0.51 - 4.34) | 0.47 |
| rs9527029 | C/C | Ref | - | - | Ref |  | - |
|  | C/T | 1.65 | (0.55 - 4.95) | 0.37 | 2.15 | (0.49 - 9.34) | 0.31 |
|  | T/T | 0.93 | (0.67 - 1.28) | 0.64 | 1.06 | (0.65 - 1.73) | 0.81 |
| rs564481 | C/C | Ref | - | - | Ref |  | - |
|  | C/T | 1.12 | (0.80 - 1.55) | 0.51 | 0.98 | (0.56 - 1.70) | 0.94 |
|  | T/T | 1.11 | (0.86 - 1.42) | 0.43 | 1.36 | (0.93 - 2.01) | 0.11 |
| rs643780 | G/G | Ref | - | - | Ref | - | - |
|  | A/G | 1.24 | (0.95 - 1.61) | 0.12 | 1.39 | (0.90 - 2.14) | 0.13 |
|  | A/A | 1.05 | (0.44 - 2.54) | 0.91 | 1.56 | (0.46 - 5.32) | 0.48 |

OR, odds ratio; CI, confidence interval

*statistical significance

**Supplementary Table 9.** Odds ratio (95% confidence interval) of genotype for *KL* SNPs on all-cause mortality and cardiovascular mortality using multivariate logistic regression analysis with major homozygote as reference

| SNP |  | All-cause mortality | | | Cardiovascular mortality | | |
| --- | --- | --- | --- | --- | --- | --- | --- |
|  | Allele | Adjusted OR | (95% CI) | *P* value | Adjusted OR | (95% CI) | *P* value |
| rs398655 | C/C | Ref | - | - | Ref |  | - |
|  | C/A | 1.04 | 0.76 - 1.42 | 0.83 | 1.10 | 0.68 - 1.78 | 0.69 |
|  | A/A | 1.07 | 0.81 - 1.41 | 0.62 | 0.92 | 0.59 - 1.44 | 0.72 |
| rs562020 | G/G | Ref |  | - | Ref |  | - |
|  | G/A | 1.16 | 0.90 - 1.49 | 0.25 | 1.42 | 0.95 - 2.12 | 0.084 |
|  | A/A | 0.93 | 0.61 - 1.42 | 0.75 | 1.07 | 0.55 - 2.08 | 0.84 |
| rs495392 | C/C | Ref |  | - | Ref |  | - |
|  | C/A | 0.79 | 0.62 - 1.01 | 0.07 | 0.66 | 0.44 - 0.97 | 0.036* |
|  | A/A | 0.77 | 0.46 - 1.28 | 0.32 | 0.74 | 0.34 - 1.61 | 0.45 |
| rs385564 | C/C | Ref | - | - | Ref | - | - |
|  | G/C | 1.03 | 0.68 - 1.55 | 0.89 | 0.81 | 0.41 - 1.59 | 0.53 |
|  | G/G | 0.95 | 0.74 - 1.23 | 0.72 | 1.19 | 0.80 - 1.77 | 0.39 |
| rs499091 | A/A | Ref | - | - | Ref | - | - |
|  | G/A | 1.14 | 0.79 - 1.63 | 0.48 | 1.04 | 0.57 - 1.91 | 0.89 |
|  | G/G | 1.20 | 0.92 - 1.57 | 0.17 | 1.40 | 0.93 - 2.11 | 0.11 |
| rs211234 | G/G | Ref | - | - | Ref | - | - |
|  | A/G | 0.90 | 0.69 - 1.17 | 0.43 | 0.82 | 0.55 - 1.22 | 0.32 |
|  | A/A | 1.01 | 0.72 - 1.43 | 0.95 | 0.66 | 0.35 - 1.25 | 0.20 |
| rs505143 | G/G | Ref | - | - | Ref | - | - |
|  | A/G | 1.22 | 0.93 - 1.58 | 0.15 | 1.31 | 0.88 - 1.96 | 0.19 |
|  | A/A | 1.20 | 0.84 - 1.72 | 0.31 | 0.93 | 0.49 - 1.74 | 0.81 |
| rs563925 | C/C | Ref | - | - | Ref | - | - |
|  | A/C | 0.93 | 0.72 - 1.20 | 0.58 | 0.96 | 0.65 - 1.43 | 0.85 |
|  | A/A | 0.75 | 0.49 - 1.15 | 0.19 | 1.00 | 0.52 - 1.89 | 0.99 |
| rs9526981 | T/T | Ref | - | - | Ref | - | - |
|  | G/T | 0.93 | 0.66 - 1.32 | 0.69 | 0.98 | 0.58 - 1.66 | 0.95 |
|  | G/G | 1.62 | 0.46 - 5.77 | 0.45 | 2.54 | 0.50 - 12.9 | 0.26 |
| rs577912 | G/G | Ref | - | - | Ref | - | - |
|  | T/G | 0.91 | 0.68 - 1.20 | 0.49 | 0.82 | 0.52 - 1.28 | 0.38 |
|  | T/T | 0.48 | 0.15 - 1.55 | 0.22 | 1.07 | 0.28 - 4.07 | 0.93 |
| rs2320762 | T/T | Ref | - | - | Ref | - | - |
|  | G/T | 1.08 | 0.84 - 1.40 | 0.55 | 1.11 | 0.73 - 1.70 | 0.62 |
|  | G/G | 0.84 | 0.58 - 1.22 | 0.36 | 1.11 | 0.64 - 1.94 | 0.71 |
| rs9536282 | C/C | Ref | - | - | Ref |  | - |
|  | C/T | 0.99 | 0.75 - 1.30 | 0.92 | 1.01 | 0.66 - 1.56 | 0.96 |
|  | T/T | 1.52 | 0.69 - 3.33 | 0.30 | 1.49 | 0.41 - 5.37 | 0.54 |
| rs9536314 | T/T | Ref | - | - | Ref | - | - |
|  | G/T | 1.02 | 0.77 - 1.34 | 0.91 | 1.05 | 0.68 - 1.61 | 0.83 |
|  | G/G | 1.52 | 0.69 - 3.31 | 0.30 | 1.53 | 0.43 - 5.48 | 0.51 |
| rs9527025 | G/G | Ref | - | - | Ref | - | - |
|  | C/G | 0.98 | 0.74 - 1.29 | 0.86 | 0.97 | 0.63 - 1.50 | 0.90 |
|  | C/C | 1.56 | 0.69 - 3.50 | 0.29 | 1.52 | 0.41 - 5.66 | 0.53 |
| rs9527026 | G/G | Ref | - | - | Ref | - | - |
|  | A/G | 0.99 | 0.75 - 1.30 | 0.94 | 0.99 | 0.64 - 1.52 | 0.95 |
|  | A/A | 1.55 | 0.69 - 3.48 | 0.29 | 1.49 | 0.40 - 5.58 | 0.55 |
| rs9527029 | C/C | Ref | - | - | Ref |  | - |
|  | C/T | 1.62 | 0.46 - 5.74 | 0.46 | 2.62 | 0.51 - 13.4 | 0.25 |
|  | T/T | 0.92 | 0.65 - 1.30 | 0.63 | 1.02 | 0.60 - 1.72 | 0.95 |
| rs564481 | C/C | Ref | - | - | Ref |  | - |
|  | C/T | 1.22 | 0.86 - 1.73 | 0.26 | 1.07 | 0.57 - 1.98 | 0.84 |
|  | T/T | 1.11 | 0.85 - 1.45 | 0.44 | 1.46 | 0.96 - 2.20 | 0.07 |
| rs643780 | G/G | Ref | - | - | Ref | - | - |
|  | A/G | 1.27 | 0.96 - 1.68 | 0.10 | 1.63 | 1.01 - 2.63 | 0.04* |
|  | A/A | 0.85 | 0.32 - 2.28 | 0.75 | 1.21 | 0.28 - 5.20 | 0.79 |

OR, odds ratio; CI, confidence interval

Adjusted odds ratio: adjusted for age, body mass index, smoking, comorbidities (hypertension, diabetes, coronary artery disease, stroke, cancer), estimated glomerular filtration rate, phosphate, and Fibroblast growth factor 23

*statistical significance

**Supplementary Table 10.** Logistic regression analysis to estimate the risk of all-cause mortality associated with genetic variation in the *KL* gene in Swedish elderly males based on the dominant model and recessive models

|  |  | Dominant model | | | | Recessive model | | | |
| --- | --- | --- | --- | --- | --- | --- | --- | --- | --- |
| SNP | Alleles | Crude OR (95% CI) | *P* | Adjusted OR (95% CI) | *P* | Crude OR (95% CI) | *P* | Adjusted OR (95% CI) | *P* |
| rs398655 | C/A | 0.98 (0.74 - 1.29) | 0.87 | 1.01 (0.75 - 1.36) | 0.96 | 0.93 (0.73 - 1.18) | 0.56 | 0.94 (0.73 - 1.22) | 0.65 |
| rs562020 | G/A | 0.95 (0.76 - 1.20) | 0.69 | 0.90 (0.70 - 1.14) | 0.38 | 1.18 (0.80 - 1.73) | 0.40 | 1.16 (0.77 - 1.74) | 0.49 |
| rs495392 | C/A | 0.88 (0.70 - 1.10) | 0.26 | 0.82 (0.64 - 1.04) | 0.11 | 1.18 (0.74 - 1.88) | 0.49 | 1.13 (0.69 - 1.85) | 0.62 |
| rs385564 | C/G | 1.00 (0.80 - 1.26) | 0.97 | 0.97 (0.76 - 1.23) | 0.80 | 1.12 (0.77 - 1.63) | 0.54 | 1.05 (0.71 - 1.56) | 0.80 |
| rs499091 | A/G | 1.13 (0.89 - 1.43) | 0.32 | 1.19 (0.92 - 1.53) | 0.19 | 0.91 (0.67 - 1.24) | 0.57 | 1.02 (0.74 - 1.41) | 0.90 |
| rs211234 | G/A | 0.92 (0.73 - 1.16) | 0.48 | 0.90 (0.70 - 1.15) | 0.39 | 1.03 (0.76 - 1.41) | 0.83 | 0.94 (0.68 - 1.31) | 0.73 |
| rs505143 | G/A | 0.85 (0.67 - 1.07) | 0.17 | 0.82 (0.64 - 1.06) | 0.13 | 1.02 (0.75 - 1.38) | 0.92 | 0.93 (0.67 - 1.29) | 0.67 |
| rs563925 | C/A | 0.99 (0.79 - 1.24) | 0.91 | 0.98 (0.77 - 1.25) | 0.89 | 1.34 (0.90 - 2.00) | 0.15 | 1.29 (0.85 - 1.95) | 0.24 |
| rs9526981 | T/G | 1.03 (0.76 - 1.42) | 0.83 | 1.04 (0.75 - 1.46) | 0.80 | 0.64 (0.22 - 1.90) | 0.42 | 0.61 (0.17 - 2.18) | 0.45 |
| rs577912 | G/T | 0.88 (0.68 - 1.14) | 0.34 | 0.88 (0.67 - 1.15) | 0.35 | 0.67 (0.24 - 1.88) | 0.45 | 0.49 (0.15 - 1.63) | 0.25 |
| rs2320762 | T/G | 1.01 (0.81 - 1.28) | 0.90 | 0.98 (0.77 - 1.26) | 0.89 | 1.21 (0.88 - 1.67) | 0.24 | 1.24 (0.88 - 1.75) | 0.21 |
| rs9536282 | C/T | 1.05 (0.82 - 1.34) | 0.70 | 1.02 (0.78 - 1.33) | 0.90 | 1.59 (0.91- 2.79) | 0.10 | 1.18 (0.63 - 2.23) | 0.60 |
| rs9536314 | T/G | 0.92 (0.72 - 1.18) | 0.52 | 0.95 (0.73 - 1.25) | 0.73 | 0.78 (0.55- 1.08) | 0.14 | 0.85 (0.58 - 1.25) | 0.42 |
| rs9527025 | G/C | 0.92 (0.72 - 1.18) | 0.53 | 0.94 (0.72 - 1.23) | 0.67 | 0.49 (0.25 - 0.93) | 0.03* | 0.63 (0.29 - 1.39) | 0.25 |
| rs9527026 | G/A | 0.93 (0.73 - 1.19) | 0.57 | 0.96 (0.73 - 1.25) | 0.75 | 0.51 (0.27 - 0.98) | 0.04* | 0.64 (0.29 - 1.41) | 0.27 |
| rs9527029 | C/T | 0.96 (0.70 - 1.32) | 0.79 | 0.94 (0.67 - 1.32) | 0.73 | 1.67 (0.56 - 5.00) | 0.36 | 1.64 (0.46 - 5.86) | 0.45 |
| rs564481 | C/T | 1.11 (0.88 - 1.40) | 0.39 | 1.14 (0.89 - 1.46) | 0.31 | 1.05 (0.78 - 1.42) | 0.72 | 1.15 (0.84 - 1.58) | 0.38 |
| rs643780 | G/A | 1.23 (0.95 - 1.60) | 0.11 | 1.28 (0.97 - 1.69) | 0.08 | 1.12 (0.47 - 2.64) | 0.80 | 1.41 (0.54 - 3.66) | 0.48 |

Adjusted odds ratio: adjusted for age, body mass index, smoking, comorbidities (hypertension, diabetes, coronary artery disease, stroke, cancer), estimated glomerular filtration rate, phosphate, and Fibroblast growth factor 23

*statistical significance

**Supplementary Table 11.** Logistic regression analysis to estimate the risk of cardiovascular mortality associated with genetic variation in the *KL* gene in Swedish elderly males based on the dominant model and recessive models

|  |  | Dominant model | | | | Recessive model | | | |
| --- | --- | --- | --- | --- | --- | --- | --- | --- | --- |
| SNP | Alleles | Crude OR (95% CI) | *P* | Adjusted OR (95% CI) | *P* | Crude OR (95% CI) | *P* | Adjusted OR (95% CI) | *P* |
| rs398655 | C/A | 1.18 (0.78 - 1.78) | 0.42 | 1.14 (0.72 - 1.79) | 0.58 | 1.15 (0.79 - 1.69) | 0.47 | 1.12 (0.74 - 1.69) | 0.60 |
| rs562020 | G/A | 0.80 (0.57 - 1.14) | 0.21 | 0.74 (0.50 - 1.08) | 0.12 | 1.12 (0.63 - 2.02) | 0.69 | 1.13 (0.60 - 2.12) | 0.71 |
| rs495392 | C/A | 0.78 (0.55 -1.10) | 0.15 | 0.69 (0.47 - 1.00) | 0.05 | 1.05 (0.53 - 2.10) | 0.88 | 1.07 (0.51 - 2.21) | 0.86 |
| rs385564 | C/G | 1.04 (0.74 - 1.47) | 0.81 | 1.11 (0.76 - 1.62) | 0.58 | 0.75 (0.39 - 1.45) | 0.39 | 0.74 (0.37 - 1.47) | 0.39 |
| rs499091 | A/G | 1.25 (0.87 - 1.82) | 0.23 | 1.31 (0.88 - 1.96) | 0.19 | 0.83 (0.51 - 1.34) | 0.44 | 0.85 (0.50 - 1.46) | 0.56 |
| rs211234 | G/A | 0.92 (0.64 - 1.32) | 0.63 | 0.89 (0.60 - 1.31) | 0.55 | 1.39 (0.81 - 2.36) | 0.23 | 1.39 (0.76 - 2.53) | 0.29 |
| rs505143 | G/A | 0.84 (0.59 - 1.21) | 0.36 | 0.82 (0.56 - 1.21) | 0.32 | 1.29 (0.78 - 2.13) | 0.33 | 1.26 (0.72 - 2.23) | 0.42 |
| rs563925 | C/A | 1.00 (0.71 - 1.40) | 0.98 | 0.96 (0.66 - 1.40) | 0.84 | 1.13 (0.63 - 2.03) | 0.67 | 0.98 (0.54 - 1.80) | 0.96 |
| rs9526981 | T/G | 0.93 (0.58 - 1.49) | 0.76 | 0.96 (0.58 - 1.58) | 0.86 | 0.51 (0.12 - 2.19) | 0.36 | 0.39 (0.08 - 1.87) | 0.24 |
| rs577912 | G/T | 0.93 (0.63 - 1.38) | 0.71 | 0.83 (0.54 - 1.28) | 0.39 | 1.38 (0.42 - 4.51) | 0.59 | 1.12 (0.26 - 4.80) | 0.88 |
| rs2320762 | T/G | 0.96 (0.67 - 1.37) | 0.83 | 0.90 (0.61 - 1.33) | 0.59 | 1.03 (0.64 - 1.65) | 0.91 | 0.96 (0.58 - 1.58) | 0.86 |
| rs9536282 | C/T | 1.06 (0.73 - 1.55) | 0.75 | 1.04 (0.69 - 1.58) | 0.85 | 2.08 (0.92 - 4.67) | 0.08 | 2.08( 0.79 - 5.44) | 0.14 |
| rs9536314 | T/G | 0.90 (0.62 - 1.32) | 0.59 | 0.93 (0.61 - 1.41) | 0.73 | 0.79 (0.48 - 1.32) | 0.37 | 0.90 (0.48 - 1.66) | 0.73 |
| rs9527025 | G/C | 0.93 (0.64 - 1.37) | 0.72 | 0.94 (0.62 - 1.43) | 0.79 | 0.62 (0.22 - 1.75) | 0.37 | 0.65 (0.19 - 2.21) | 0.49 |
| rs9527026 | G/A | 0.94 (0.64 - 1.37) | 0.74 | 0.96 (0.63 - 1.45) | 0.84 | 0.66 (0.23 - 1.85) | 0.43 | 0.66 (0.19 - 2.27) | 0.51 |
| rs9527029 | C/T | 1.11 (0.70 - 1.78) | 0.65 | 1.08 (0.65 - 1.79) | 0.76 | 2.13 (0.49 - 9.24) | 0.31 | 2.61 (0.55 - 12.48) | 0.23 |
| rs564481 | C/T | 1.26 (0.87 - 1.83) | 0.22 | 0.39 (0.08 - 1.87) | 0.24 | 0.81 (0.50 - 1.33) | 0.41 | 0.96 (0.58 - 1.58) | 0.86 |
| rs643780 | G/A | 1.34 (0.89 - 2.03) | 0.17 | 1.61 (1.02 - 2.54) | 0.04* | 0.83 (0.25 - 2.68) | 0.75 | 1.20 (0.28 - 5.16) | 0.81 |

Adjusted odds ratio: adjust for age, body mass index, smoking, comorbidities (hypertension, diabetes, coronary artery disease, stroke, cancer), estimated glomerular filtration rate, phosphate, and Fibroblast growth factor 23

*statistical significance

**Supplementary Table 12.** Hazard ratio (95% confidence interval) of genotype for tagging SNPs for all-cause mortality and cardiovascular mortality using univariate and multivariate Cox regression analysis with major homozygote as reference

| SNP |  | Unadjusted model | | | Model 1 | | | Model 2 | | | Model 3 | | |
| --- | --- | --- | --- | --- | --- | --- | --- | --- | --- | --- | --- | --- | --- |
| SNP | Allele | Crude HR | (95% CI) | *P* value | Adjusted HR | (95% CI) | *P* value | Adjusted HR | (95% CI) | *P* value | Adjusted HR | (95% CI) | *P* value |
| All-cause mortality | | | | | | | | | | | | | |
| rs9536282 | C/C | Ref | - | - | Ref | - | - | Ref | - | - | Ref | - | - |
|  | C/T | 0.96 | (0.75 - 1.22) | 0.73 | 0.98 | (0.77 - 1.25) | 0.84 | 0.97 | (0.76 - 1.24) | 0.83 | 0.98 | (0.77 - 1.25) | 0.88 |
|  | T/T | 1.69 | (0.95 - 3.02) | 0.07 | 1.67 | (0.94 - 2.99) | 0.08 | 1.66 | (0.93 - 2.97) | 0.09 | 1.68 | (0.94 - 3) | 0.08 |
| rs9536314 | T/T | Ref | - | - | Ref | - | - | Ref | - | - | Ref | - | - |
|  | G/T | 0.99 | (0.78 - 1.27) | 0.96 | 1.01 | (0.79 - 1.29) | 0.93 | 1.01 | (0.79 - 1.29) | 0.94 | 1.02 | (0.8 - 1.3) | 0.89 |
|  | G/G | 1.72 | (0.96 - 3.07) | 0.07 | 1.69 | (0.95 - 3.02) | 0.08 | 1.69 | (0.95 - 3.01) | 0.08 | 1.70 | (0.95 - 3.03) | 0.07 |
| rs9527025 | G/G | Ref | - | - | Ref | - | - | Ref | - | - | Ref | - | - |
|  | C/G | 1.02 | (0.80 - 1.29) | 0.90 | 1.00 | (0.78 - 1.27) | 0.99 | 1.00 | (0.79 - 1.27) | 1.00 | 0.99 | (0.78 - 1.26) | 0.95 |
|  | C/C | 1.82 | (0.998 - 3.33) | 0.05 | 1.75 | (0.96 - 3.19) | 0.07 | 1.74 | (0.95 - 3.17) | 0.07 | 1.74 | (0.95 - 3.18) | 0.07 |
| rs643780 | G/G | Ref | - | - | Ref | - | - | Ref | - | - | Ref | - | - |
|  | G/A | 1.22 | (0.95 - 1.57) | 0.12 | 1.25 | (0.97 - 1.61) | 0.08 | 1.25 | (0.97 - 1.6) | 0.08 | 1.24 | (0.96 - 1.59) | 0.10 |
|  | A/A | 1.08 | (0.47 - 2.48) | 0.85 | 1.10 | (0.48 - 2.53) | 0.82 | 1.10 | (0.48 - 2.51) | 0.83 | 1.09 | (0.48 - 2.5) | 0.84 |
| Cardiovascular mortality | | | | | | | | | | | | | |
| rs9536282 | C/C | Ref |  | - | Ref | - | - | Ref | - | - | Ref | - | - |
|  | C/T | 1.00 | (0.68 - 1.47) | 0.99 | 1.03 | (0.7 - 1.52) | 0.88 | 1.03 | (0.7 - 1.51) | 0.89 | 1.04 | (0.71 - 1.53) | 0.85 |
|  | T/T | 1.47 | (0.54 - 3.99) | 0.45 | 1.44 | (0.53 - 3.9) | 0.48 | 1.42 | (0.52 - 3.87) | 0.49 | 1.43 | (0.53 - 3.88) | 0.48 |
| rs9536314 | T/T | Ref | - | - | Ref | - | - | Ref | - | - | Ref | - | - |
|  | G/T | 1.05 | (0.71 - 1.55) | 0.80 | 1.08 | (0.73 - 1.58) | 0.71 | 1.07 | (0.73 - 1.58) | 0.72 | 1.08 | (0.74 - 1.6) | 0.68 |
|  | G/G | 1.52 | (0.56 - 4.14) | 0.41 | 1.48 | (0.55 - 4.03) | 0.44 | 1.48 | (0.54 - 4.01) | 0.45 | 1.48 | (0.55 - 4.04) | 0.44 |
| rs9527025 | G/G | Ref | - | - | Ref | - | - | Ref | - | - | Ref | - | - |
|  | C/G | 0.99 | (0.68 - 1.46) | 0.97 | 0.97 | (0.66 - 1.42) | 0.87 | 0.97 | (0.66 - 1.43) | 0.88 | 0.96 | (0.65 - 1.41) | 0.84 |
|  | C/C | 1.54 | (0.55 - 4.33) | 0.41 | 1.44 | (0.51 - 4.07) | 0.49 | 1.44 | (0.51 - 4.05) | 0.49 | 1.43 | (0.51 - 4.03) | 0.50 |
| rs643780 | G/G | Ref | - | - | Ref | - | - | Ref | - | - | Ref | - | - |
|  | G/A | 1.39 | (0.91 - 2.12) | 0.13 | 1.43 | (0.94 - 2.19) | 0.10 | 1.43 | (0.94 - 2.18) | 0.10 | 1.41 | (0.93 - 2.16) | 0.11 |
|  | A/A | 1.57 | (0.48 - 5.16) | 0.46 | 1.62 | (0.49 - 5.33) | 0.43 | 1.61 | (0.49 - 5.31) | 0.43 | 1.60 | (0.49 - 5.28) | 0.44 |

Model 1: adjusted for age

Model 2: adjusted for age and body mass index,

Model 3: adjusted for age, body mass index and smoking

Reference List

1. Kumar P, Henikoff S, Ng PC. Predicting the effects of coding non-synonymous variants on protein function using the SIFT algorithm. *Nat Protoc.* 2009;4(7):1073-1081.

2. Adzhubei I, Jordan DM, Sunyaev SR. Predicting functional effect of human missense mutations using PolyPhen-2. *Curr Protoc Hum Genet.* 2013;Chapter 7:Unit7 20.

3. Choi Y, Chan AP. PROVEAN web server: a tool to predict the functional effect of amino acid substitutions and indels. *Bioinformatics.* 2015;31(16):2745-2747.

4. Yue P, Melamud E, Moult J. SNPs3D: candidate gene and SNP selection for association studies. *BMC Bioinformatics.* 2006;7:166.

5. Karchin R, Diekhans M, Kelly L, et al. LS-SNP: large-scale annotation of coding non-synonymous SNPs based on multiple information sources. *Bioinformatics.* 2005;21(12):2814-2820.

6. Li B, Krishnan VG, Mort ME, et al. Automated inference of molecular mechanisms of disease from amino acid substitutions. *Bioinformatics.* 2009;25(21):2744-2750.

7. Kircher M, Witten DM, Jain P, O'Roak BJ, Cooper GM, Shendure J. A general framework for estimating the relative pathogenicity of human genetic variants. *Nat Genet.* 2014;46(3):310-315.

8. Quang D, Chen Y, Xie X. DANN: a deep learning approach for annotating the pathogenicity of genetic variants. *Bioinformatics.* 2015;31(5):761-763.

9. Shihab HA, Rogers MF, Gough J, et al. An integrative approach to predicting the functional effects of non-coding and coding sequence variation. *Bioinformatics.* 2015;31(10):1536-1543.

10. Fu Y, Liu Z, Lou S, et al. FunSeq2: a framework for prioritizing noncoding regulatory variants in cancer. *Genome Biol.* 2014;15(10):480.

11. Ritchie GR, Dunham I, Zeggini E, Flicek P. Functional annotation of noncoding sequence variants. *Nat Methods.* 2014;11(3):294-296.

12. Bendl J, Musil M, Stourac J, Zendulka J, Damborsky J, Brezovsky J. PredictSNP2: A Unified Platform for Accurately Evaluating SNP Effects by Exploiting the Different Characteristics of Variants in Distinct Genomic Regions. *PLoS Comput Biol.* 2016;12(5):e1004962.

13. Capriotti E, Fariselli P. PhD-SNPg: a webserver and lightweight tool for scoring single nucleotide variants. *Nucleic Acids Res.* 2017;45(W1):W247-W252.

14. Boyle AP, Hong EL, Hariharan M, et al. Annotation of functional variation in personal genomes using RegulomeDB. *Genome Res.* 2012;22(9):1790-1797.
